# Specific reaction conditions for efficient automated ^68^Ga-radiolabeling of the FAP-2286 pseudopeptide on a GAIA^®^ synthesizer

**DOI:** 10.3389/fmed.2025.1628158

**Published:** 2025-07-22

**Authors:** Maissa Ammour, Jade Torchio, Stéphane C. Renaud, Léa Rubira, Cyril Fersing

**Affiliations:** ^1^Department of Nuclear Medicine, Institut régional du Cancer de Montpellier (ICM), University of Montpellier, Montpellier, France; ^2^IBMM, Univ Montpellier, CNRS, ENSCM, Montpellier, France

**Keywords:** radiopharmaceuticals, automated radiolabeling, gallium-68, FAP-2286, PET imaging, tumor microenvironment

## Abstract

**Introduction:**

Automated radiolabeling of gallium-68-labeled experimental radiopharmaceuticals is crucial for ensuring high reproducibility and regulatory compliance in clinical settings. FAP-2286, a promising DOTA-pseudopeptide targeting the tumor microenvironment, has demonstrated superior tumor retention compared to quinoline-based analogs, making it an attractive theranostic agent. This study aimed to optimize and automate the preparation of [^68^Ga]Ga-FAP-2286 on the GAIA^®^ synthesizer, ensuring high radiochemical purity (RCP) and radiochemical yield (RCY).

**Methods:**

Manual radiolabeling assays were initially performed to identify optimal reaction conditions, varying buffer, antioxidant, vector amount, heating time, and purification methods. The selected conditions were then adapted to an automated protocol using a GAIA^®^ module. A strong cation exchange (SCX) cartridge for ^68^Ga pre-concentration and a solid-phase extraction (SPE) step for final purification were included in the process. RCY, RCP, and stability over 4 h were assessed using radio-HPLC and radio-TLC. Additionally, the applicability of the optimized automated method was evaluated for 3BP-3940, a structurally related pseudopeptide.

**Results:**

Initial optimization studies identified sodium acetate buffer 0.1 M with methionine as an antioxidant, 25 μg of FAP-2286, and a 4-min heating time as the best manual radiolabeling conditions, achieving a RCP > 98%. In the automated synthesis, adjustments were made, including doubling the vector amount and extending heating to 9 min, resulting over three test-batches in a moderate RCY of 59.85 ± 3.73% and a RCP just over 94% up to 4 h after the end of synthesis. Importantly, the method was successfully transposed to [^68^Ga]Ga-3BP-3940, yielding better RCY (75.62 ± 11.76%), RCP and stability profiles (> 95.95% over 4 h).

**Conclusion:**

This study established a robust, automated protocol for the synthesis of [^68^Ga]Ga-FAP-2286, ensuring high purity, reproducibility, and compatibility with clinical applications. The method’s successful adaptation to 3BP-3940 highlights its versatility for such radiopharmaceuticals, supporting the broader implementation of automated theranostic agent production in nuclear medicine.

## 1 Introduction

Positron emission tomography (PET) imaging is now an established and essential tool in oncology, helping in diagnosis, staging and assessment of treatment response in many types of cancer (1). Beyond its more common clinical applications, PET imaging is also a highly dynamic field of research and development (2, 3), particularly with the increasing availability of gallium-68 (^68^Ga) (4). The widespread use of ^68^Ga is largely attributed to the convenience of its generator-based production and the possibility of radiolabeling a wide variety of targeting molecules, provided that they are functionalized with an appropriate chelating agent, e.g., 2,2′,2″,2″-(1,4,7,10-tetraazacyclododecane-1,4,7,10-tetrayl) tetraacetic acid (DOTA), 2-(4,7-bis (carboxymethyl)-1,4,7-triazonan-1-yl) pentanedioic acid (NODAGA), or 2,2′,2″-(1,4,7-triazacyclononane-1,4,7-triyl) triacetic acid (NOTA) (5). As a result, such a framework tends to facilitate the rapid translation of novel radiopharmaceuticals into clinical applications.

**FIGURE 1:**
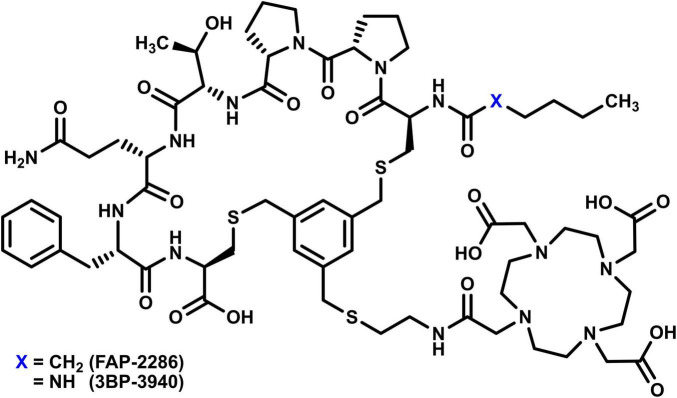
Chemical structure of the vector molecules FAP-2286 and 3BP-3940.

In recent years, there has been growing interest in targeting the tumor microenvironment, particularly through fibroblast activation protein (FAP) inhibitors (FAPIs) (6). The first generation of FAPI molecules, sharing a quinoline moiety and a glycine-cyanoproline motif, was developed as PET imaging probes radiolabeled with ^68^Ga (7–9). Among these compounds, FAPI-04 and FAPI-46 are widely used in clinical practice, notably for cancers where the non-specific but highly sensitive [^18^F] FDG radiopharmaceutical fails to provide satisfactory imaging results (10, 11). A second generation of FAPI compounds has since been developed, featuring a pseudopeptide structure composed of a seven-amino acid sequence cyclized via reaction with 1,3,5-tris (bromomethyl) benzene to form a mesityl cyclic core, subsequently functionalized with a DOTA chelator (Figure 1). The first of these pseudopeptide FAPI compounds to be used in humans for PET imaging was FAP-2286 (rofapitide tetraxetan) (12). After an earlier study demonstrated its potent affinity for human FAP protein and its effective binding *in vitro* (IC_50_ from 1.3 to 2.2 nM) (13, 14), it was suggested that [^68^Ga]Ga-FAP-2286 was superior to [^18^F]FDG for detecting lesions in selected cancers, such as gastric, pancreatic, and hepatic tumors (particularly intrahepatic cholangiocarcinomas) (15). Subsequent studies have consolidated these results (16, 17), extending the potential applications of [^68^Ga]Ga-FAP-2286 to other tumor types such as urothelial (18, 19) and lung cancers (20). Shortly afterward, a compound directly related to FAP-2286 called 3BP-3940 was studied in clinical settings and also displayed excellent properties as a molecular PET imaging agent, including a remarkably high tumor-to-background ratio and minimal renal accumulation (21, 22). Importantly, a major advantage of anti-FAP pseudopeptide derivatives over quinoline compounds is their higher intratumoral retention (23, 24), making them suitable as theranostic vectors—enabling PET imaging when radiolabeled with ^68^Ga and therapeutic applications when radiolabeled with ^177^Lu, for example. Consequently, several studies have reported on the use and efficacy of [^177^Lu]Lu-FAP-2286 in various cancers (25–27), with case reports further supporting these findings (28–33). The FAP-2286 vector associated with the ^68^Ga/^177^Lu theranostic pair is currently being investigated in the LuMIERE phase 1/2 trial to evaluate its safety, pharmacokinetics, and preliminary efficacy in patients with selected advanced solid tumors (NCT04939610) (34).

The automation of radiopharmaceuticals production has played an increasing role in ensuring consistent and reproducible radiolabeling processes (35). While automation has long been established in the industrial production of fluorine-18-labeled compounds (36), the past two decades have seen a proliferation of synthesizers designed for nuclear medicine departments. Initially semi-automated, these systems have now evolved into fully automated platforms compatible with radiolabeling using various isotopes, including radiometals such as ^68^Ga (37, 38). Moreover, the well-documented chemistry and on-site availability of ^68^Ga make it particularly suited for the automated synthesis of experimental ^68^Ga-labeled radiopharmaceuticals using customized protocols (39, 40). By carefully selecting the nature and concentration of reaction components, radiolabeling conditions can be finely tuned to one specific vector molecule.

In this context, we investigated the optimization of ^68^Ga-radiolabeling conditions for FAP-2286 through a systematic screening of reaction buffers, antioxidant compounds, vector amounts, heating times, and purification methods. Following a series of manual radiolabeling experiments, the optimal conditions for [^68^Ga]Ga-FAP-2286 synthesis were implemented on a specific automated synthesis module (GAIA^®^, Elysia Raytest). Additionally, we explored the applicability of these optimized conditions to the radiolabeling of 3BP-3940, a pseudopeptidic compound structurally related to FAP-2286.

## 2 Materials and methods

### 2.1 Reagents and equipment

All reagents used for radiolabeling were of the highest available purity and sourced from Merck (Germany). Pharmaceutical-grade water for injection (WFI; Eau pour prép. injectables 10 mL PROAMP^®^, Aguettan, France; 100 mL Ecoflac, B. Braun, France) and 0.9% sodium chloride solution (Chlorure de sodium PROAMP^®^ 0.9% 10 mL, Aguettan, France) were employed in the process. Ethanol absolute (≥ 99.8%, Ph. Eur. grade, VWR, United States), was also used. The radiolabeling optimization experiments were carried out with non-GMP grade FAP-2286 (MedChem Express, NJ, United States). A stock solution of 1 mg/mL pseudopeptide in WFI was prepared, aliquoted into 25 μg/25 μL fractions using Eppendorf Protein LoBind Tubes (1.5 mL), and stored at −20°C for up to 3 months. Gallium-68 was eluted in 0.1 N hydrochloric acid as [^68^Ga]GaCl_3_ from a pharmaceutical-grade ^68^Ge/^68^Ga generator (GALLI AD^®^ 1.85 GBq, Ire Elit, Belgium). Consecutive elutions were spaced by a minimum of 4 h and did not exceed a 24-h interval. The manual preparation of [^68^Ga]Ga-FAP-2286 took place in a shielded cell (MEDI 2000, LemerPax, France), where both the ^68^Ge/^68^Ga generator and a dry bath (Zinsser Analytic, Germany) were housed. Automated preparation of [^68^Ga]Ga-FAP-2286 and [^68^Ga]Ga-3BP-3940 was performed on a GAIA^®^ synthesizer (Elysia-Raytest, Germany) in a shielded, GMP ISO 5 cell with laminar airflow (MEDI 9000 Research 4R, LemerPax, France) where the synthesis module and a second GALLI AD^®^ generator were positioned.

### 2.2 Manual radiolabeling assays for the study of reaction conditions

For each radiolabeling condition tested, three identical reactions were run simultaneously. Typically, aliquots of vector (1 mg/mL, i.e., 0.68 μmol/L, 12.5–50 μL) contained in 1.5 mL Eppendorf vials were warmed to room temperature and diluted in 375 μL of buffer solution. Depending on the conditions tested, 25 μL of antioxidant compound solution were also added. In the shielded cell, the ^68^Ga generator was eluted into a bulk vial to obtain ∼1 mL of ^68^Ga^3+^ solution that was not further purified. Then, 267 μL (∼120 MBq) of this gallium solution were added to each Eppendorf of the triplicate. The reaction mixtures were heated in a 95°C water bath for 4, 8, or 12 min. After the reaction, the Eppendorf tubes were allowed to cool for 5 min, after which a sample was taken from each crude mixture for quality controls. During the study of reaction conditions, quality controls only included radiochemical purity (RCP) determination by radio-HPLC and pH check by indicator strip.

The radiolabeling conditions tested were essentially inspired by protocols found in the literature, or chosen to facilitate a logical, comprehensive discussion of the results (e.g., for selected buffer concentrations) (Table 1). Each triplicate varied by only a single parameter. Buffer solutions were prepared extemporaneously as 5 mL stock solutions. Importantly, the pH of these solutions was finely adjusted with ultrapure 37% HCl, so that a mixture of 375 μL buffer and 267 μL 0.1 M HCl (mimicking the ^68^Ga-eluate) would reach a pH of 3.6–3.8, ideal for such radiolabeling reaction (41). For each buffer and mixture, pH value was measured using a recently calibrated Vario^®^ pH meter (WTW^®^, Xylem, United States) equipped with a SenTix^®^ 41 pH electrode (WTW^®^, Xylem, United States).

**TABLE 1:** Buffer solutions and antioxidant compounds tested for the preparation of [^68^Ga]Ga-FAP-2286.

Buffer solution tested	References
Sodium acetate 0.1 M	(50)
Sodium acetate 0.5 M	(51)
Sodium acetate 1.5 M	(82)
Ammonium acetate 0.1 M	(83)
Ammonium acetate 0.5 M	(84)
Ammonium acetate 1.5 M	(85)
Sodium formate 0.5 M	(75)
Sodium formate 1.5 M	(86)
HEPES 0.5 M	(87)
HEPES 1.5 M	(88, 89)
**Antioxidant compound tested**	**References**
Ascorbic acid 12 mg/mL	(52)
Gentisic acid 16 mg/mL	(63)
Methionine 10 mg/mL	(53)

Antioxidant compound solutions, i.e., ascorbic acid 14 mg/mL (79.5 mM), gensitic acid 16 mg/mL (103.8 mM) and methionine 10 mg/mL (67 mM) (Table 1), were also freshly prepared as 10 mL stock solutions. For experiments involving the addition of one of these antioxidant compounds, the stability of the radiolabeling product was monitored by radio-HPLC over 4 h.

Three different amounts of vector molecule were used in the radiolabeling tests (12.5, 25, or 50 μg, i.e., 8.5, 17, or 34 nmol) in order to identify the lowest amount of FAP-2286 required to achieve good RCP. Similarly, heating times of 4, 8, and 12 min were tested to optimize preparation duration.

Finally, four solid-phase extraction cartridge models (i.e., Sep-Pak^®^ Plus Short C_18_, Oasis HLB Plus Short, Strata-X, Sep-Pak Accell Plus CM Plus Short) were each tested for final purification on a radiolabeling triplicate. For the cartridges concerned, washing was performed with 4 mL WFI after deposition of the crude reaction medium, and elution was performed with 1.5 mL 60% ethanol.

### 2.3 Application of the best reaction conditions to an automated radiolabeling protocol

**FIGURE 2:**
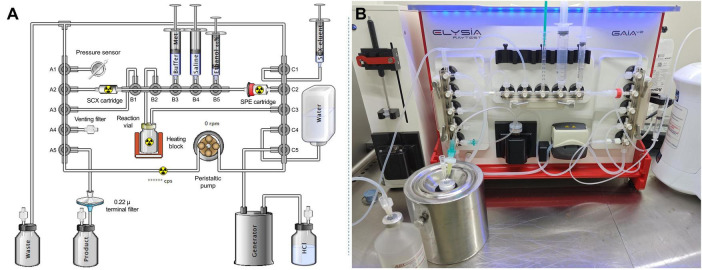
**(A)** Cassette-based scheme of the GAIA^®^ synthesizer for [^68^Ga]Ga-FAP-2286. **(B)** Photograph of the set-up on the GAIA^®^ module.

The best radiolabeling conditions resulting from manual experiments were transposed to a custom automated preparation method on the GAIA^®^ module. This system uses sterile, single-use tubing sets with 3 ramps (named A, B and C from left to right) of 5 manifolds each (numbered from 1 to 5, from top to bottom or left to right), and relies on a peristaltic pump to transfer liquids into the fluidic system. First, the cassette was assembled as shown in Figure 2. Specifically, a strong cation exchange (SCX) cartridge (Bond Elut SCX, 100 mg, 1 mL, 40 μm, Agilent) with an appropriate Luer adapter was used to connect position A2 to position B1 horizontal. Likewise, a solid phase extraction cartridge (either a Sep-Pak C_18_ Plus Short cartridge or an Oasis HLB Plus Short cartridge) was used to connect position B5 horizontal to position C2 after appropriate manual preconditioning of the cartridge with 5 mL of absolute ethanol and 5 mL of WFI. The other reagents were then connected to the manifolds, i.e., a mixture of 170 μL sodium acetate 0.8 M and 1.03 mL methionine 10 mg/mL solubilizing 50 μg vector in B3, 12.8 mL methionine 1 mg/mL in 0.9% NaCl for formulation in B4, 2.3 mL 60% ethanol for solid phase extraction (SPE) elution in vertical B5, 0.4 mL 5 M NaCl in 0.13 N HCl for SCX elution in horizontal C1, and a 500 mL bag of WFI in C4.

**FIGURE 3:**
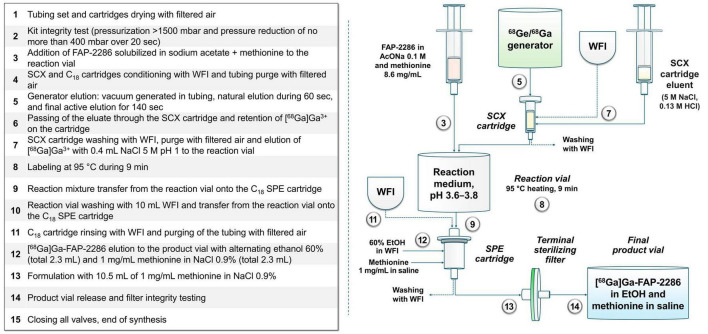
Flow chart for the automated [^68^Ga]Ga-FAP-2286 production process.

Once initiated, automated radiolabeling proceeds as follows: first, the system is purged with filtered air to remove any residual liquid from the SPE cartridge. Next, an integrity test of the tubing set is performed by pumping filtered air into the system, raising the pressure in the kit above 1,500 mbar. Once the system is sealed, the test is considered successful if the pressure drop does not exceed 400 mbar, allowing the sequence to proceed. The buffer solution and antioxidant mixture, which solubilizes the vector, is then transferred to the reaction vial. Meanwhile, the SCX and C_18_ cartridges are conditioned with WFI, and the system is purged with filtered air. At this stage, the generator can be eluted, passing the ^68^Ga-solution in 0.1 N HCl through the SCX cartridge, where ^68^Ga^3+^ ions are retained. Approximately 950 MBq were involved in the automated radiolabeling process at the time of elution from the generator. After washing with WFI, the SCX cartridge is eluted with 0.4 mL of NaCl-saturated solution in 0.13 N HCl, directing the activity to the reaction vial. The reaction vial, maintained at 60°C up to this point, is then heated to 95°C for 9 min to facilitate radiolabeling. The radiolabeled product retained on the SPE cartridge is eluted in four successive fractions of 60% ethanol (total volume: 2.3 mL), alternating with NaCl plus methionine fractions. The product solution is eluted into the product vial through a 0.22 μ sterile filter. The final product is formulated by adding the remaining 1 mg/mL methionine solution in saline (total volume: 12.8 mL), after which the terminal vial containing the product can be removed. Finally, the system performs an automated bubble point integrity test on the 0.22 μm end filter. The sequential steps of this protocol are summarized in Figure 3.

To determine the radiochemical yield (RCY) of the automated synthesis, radioactivity measurements were made in a calibrated ionization chamber (CRC^®^-25R, Capintec, United States). These measurements included the final product vial, reaction vial, waste vial, purification cartridge, and terminal filter, with activity values adjusted to the radiolabeling endpoint for accurate yield calculation. RCY is the ratio of the activity in the terminal vial to the sum of the activities of all the elements in the system, weighted by the RCP and all decay-corrected to the end of synthesis (EoS) time.

To assess the feasibility of adapting this automated radiolabeling method for another ^68^Ga-labeled pseudopeptide, the previously described protocol was applied to produce three test batches of (^68^Ga) Ga-3BP-3940, synthesized using a non-GMP grade vector (MedChem Express, NJ, United States). All parameters used in these three syntheses were identical to those employed in the automated preparation of [^68^Ga]Ga-FAP-2286.

### 2.4 Quality controls

For each radiolabeling reaction product, a RCP analysis was performed by radio-HPLC, using a Nexera X3 station (Shimadzu, Japan) supplied with HPLC-grade solvents. The apparatus included a solvent degasser (DGU-405), a solvent pump (LC40D), an autosampler (SIL-40) set at 20 μL injection volume, a column oven (CTO-40S) set at 30°C, a UV detector (SPD-40 190–700 nm) set at 254 and 280 nm and a radioactivity detector (GABI Nova with mid-energy probe and 2 × 5 μL flow cell) connected in series. A C_18_ ACE^®^ Equivalence™ column (3.0 × 150 mm, 110 Å pore size and 3 μm particles size) was used as the stationary phase. The flow rate was 0.6 mL/min and the mobile phase gradient was programmed with 0.1% TFA in water (line A) to 0.1% TFA in acetonitrile (line B) as follow: 0–1 min 95/5 A/B; 1–8 min linear gradient from 95/5 A/B to 60/40 A/B; 8–9 min 60/40 A/B; 9–10 min linear gradient from 60/40 A/B to 95/5 A/B; 10–12 min 95/5 A/B. RCP was calculated using the dedicated software (Gina X, Elysia Raytest, Germany) by spectra integration and comparison of areas under peaks.

During the radiolabeling assays, pH of the reaction products was controlled using either 2-zones Rota pH 1–11 indicator paper (VWR, PA, United States) or MQuant^®^ pH 2.5–4.5 indicator strips (Merk, NJ, Unites States).

Specific quality controls were performed only on test batches produced via the automated process:

• Radio-TLC analyses used a two-strip iTLC-SG system inspired from [^68^Ga]Ga-edotreotide summary of product characteristics (42), with aqueous ammonium acetate 1 M in methanol (1:1 mixture) (conditions A) and aqueous sodium citrate 0.1 M pH 5 (conditions B) as mobile phases. Measurement of the percentages of radioactivity at the origin and at the solvent front was carried out using a radio-TLC scanner (miniGITA^®^ Star, Elysia-Raytest, Germany). The corresponding acquisition software (TLC Control v.2.30, Raytest, Germany) and analysis software (GINA Star TLC™ v.6.0, Elysia-Raytest, Germany) were used for data analysis. Under conditions A, Rf values of 0.0–0.2 for ^68^Ga-impurities and 0.8–1.0 for [^68^Ga]Ga-FAP-2286 were expected. Under conditions B, Rf values of 0.0–0.2 for [^68^Ga]Ga-FAP-2286 and 0.8–1.0 for free ^68^Ga were expected.
• Gamma spectrometry was conducted on a low-activity sample (around 100 kBq in 1 mL) from each validation batch of [^68^Ga]Ga-FAP-2286 using a Hidex AMG^®^ gamma counter (LabLogic, United Kingdom). Identification focused on the 511 and 1,077 keV peaks of annihilation photons.
• The half-life was confirmed by performing multiple measurements over approximately 1 h. Expected values ranged between 61 and 75 min, with a theoretical reference of 67.71 min (43).
• To evaluate radionuclide purity, the same samples used for radionuclide identity testing were reanalyzed by gamma counting after 48 h of decay. This measurement allowed the identification of any residual ^68^Ga activity resulting from ^68^Ge breakthrough or other radionuclide impurities with long half-life. Residual radioactivity after 48 h was expected to remain below 0.001% of the initial activity recorded in each sample.

The RCP of the test batches was assessed by radio-HPLC for up to 4 h post-preparation as described above.

### 2.5 Statistical analysis

Student’s *t*-test was used to compare triplicate RCP or RCY values obtained under two different reaction conditions. For each triplicate tested, normal distribution of data was confirmed by a Shapiro-Wilk test. The *p*-value was used to estimate statistical significance, with *p* ≤ 0.05 considered significant. For stability tests, the relationship between variation in RCP and time was estimated by regression analysis.

## 3 Results

### 3.1 Selection of optimal radiolabeling conditions

The general process for manual radiolabeling described above allowed the efficient screening of 17 different radiolabeling conditions and four purification methods, representing more than 60 individual radiolabeling reactions.

**FIGURE 4:**
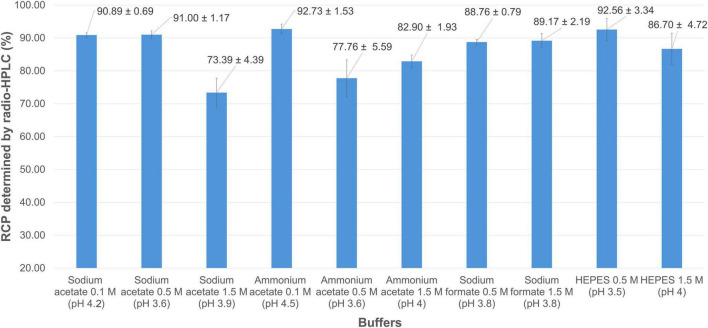
Mean RCP values (determined by radio-HPLC) for [^68^Ga]Ga-FAP-2286 using buffers of different types and molarities.

The radio-HPLC RCP values measured from different reaction buffers are summarized in Figure 4, and suggest that the preparation of [^68^Ga] Ga-FAP-2286 allows the use of a variety of buffer types and molarities. Indeed, 8 of the 10 buffers tested led to a RCP > 80% without terminal purification. This was particularly the case for sodium formate, for which molarity did not seem to have a significant influence (RCP = 88.76 ± 0.79% at 0.5 M; RCP = 89.17 ± 2.19% at 1.5 M, p = 0.778). The HEPES buffer produced comparable or even slightly improved results at the low concentration (RCP = 92.56 ± 3.34% at 0.5 M; RCP = 86.70 ± 4.72% at 1.5 M). This molecule, which belongs to Good’s buffers (44, 45), is particularly well suited for the preparation of ^68^Ga-radiopharmaceuticals due to its weak complexing properties and excellent pH control (46, 47). However, regulatory restrictions in final radiopharmaceuticals formulations make its use inadvisable whenever possible (48, 49). Excellent RCP values were achieved with acetate buffers at low concentrations (sodium acetate 0.1 M: 90.89 ± 0.69%; ammonium acetate 0.1 M: 92.73 ± 1.53%), while an increase in molarity seemed to be unfavorable to the good complexation of gallium by the DOTA-pseudopeptide (sodium acetate 1.5 M: 73.39 ± 4.39%, p = 0.024; ammonium acetate 1.5 M: 82.90 ± 1.93%, *p* = 0.023). Overall, as the RCP values obtained with low-molarity acetate buffers were not significantly different from each other (*p* = 0.161), and in view of the efficiency of sodium acetate in numerous other ^68^Ga-radiolabeling protocols (50–58), it was selected for the following assays.

**FIGURE 5:**
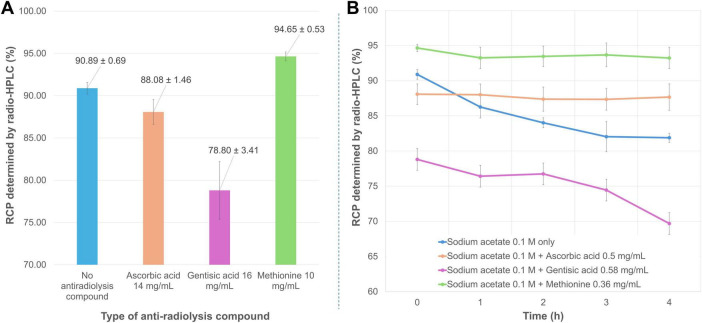
(**A**) Mean RCP values (determined by radio-HPLC) for [^68^Ga]Ga-FAP-2286 prepared in the presence of different antioxidant compounds. **(B)** Time course of mean RCP (determined by radio-HPLC) for [^68^Ga]Ga-FAP-2286 in the presence of different antioxidant compounds.

Among the three antioxidant compounds tested (Figure 5A), ascorbic acid at 79.5 mM (∼2.9 mM in the reaction volume) slightly reduced RCP (88.08 ± 1.46%), though not significantly (*p* = 0.061) when added to the radiolabeling mixture. The difference in RCP between radiolabeling with 0.1 M sodium acetate alone and with the same buffer supplemented with gentisic acid (∼3.8 mM in the reaction volume) was significantly unfavorable for this antioxidant (RCP = 78.80 ± 3.41%, *p* = 0.022). Conversely, adding methionine (∼2.4 mM in the reaction medium) to the radiolabeling reaction significantly improved purity, as measured by radio-HPLC (RCP = 94.65 ± 0.53%, *p* = 0.002). Both methionine and ascorbic acid were highly effective in maintaining RCP over 4 h (slope of regression line not significantly different from zero, *p* = 0.23 and 0.20, respectively) (Figure 5B). In the absence of an antioxidant compound, the purity of the radiolabeled product gradually decreased over time (*p* = 0.017; RCP at 4 h = 81.88 ± 0.65%). Notably, this decline was even more pronounced in the presence of gentisic acid (*p* = 0.025; RCP at 4 h = 69.68 ± 8.88%). In view of the above results, the subsequent radiolabeling tests were carried out in the presence of methionine in the reaction medium. Interestingly, modifying the reaction medium to significantly increase the amount and concentration of the antioxidant agent allowed for further optimization of the radiolabeling conditions. This was achieved by combining 60 μL of 0.8 M sodium acetate (final concentration ∼0.1 M) with 365 μL of 10 mg/mL methionine (final concentration ∼8.6 mg/mL) in a total reaction volume of 692 μL. Under these conditions, which were used for the subsequent assays, the average RCP reached 98.12 ± 0.72%.

**FIGURE 6:**
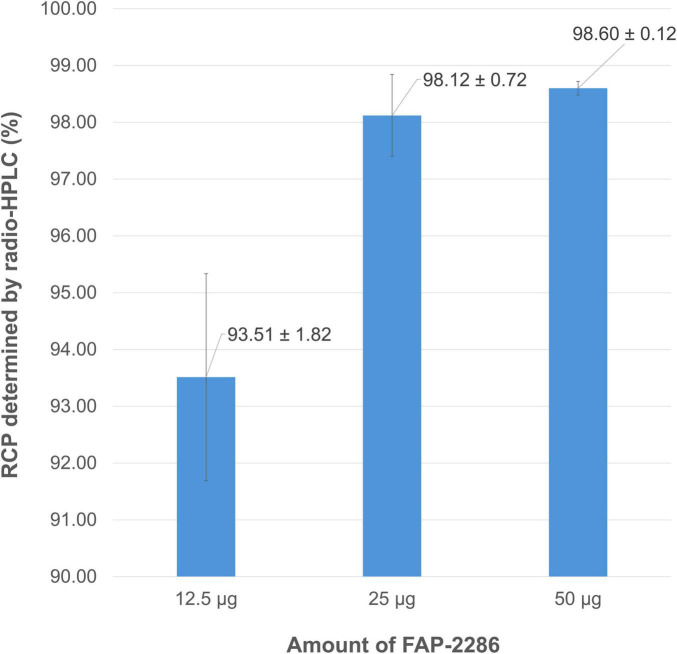
Mean RCP values (determined by radio-HPLC) for [^68^Ga]Ga-FAP-2286 depending on the amount of vector molecule used for radiolabeling.

Studying the amount of vector molecule involved in a radiolabeling reaction is crucial, as reducing this quantity can increase the molar activity of the radiopharmaceutical preparation (i.e., the amount of vectorized radioactivity per mol of vector molecule). Conversely, increasing the vector amount may enhance the complexation of ^68^Ga^3+^, following the rationale of the law of mass action. For FAP-2286, reducing the pseudopeptide quantity by half from 25 μg to 12.5 μg (i.e., from 17 nmol to 8.5 nmol) significantly decreased the purity of the radiolabeling product (RCP = 93.51 ± 1.82% vs. 98.12 ± 0.72%, *p* = 0.015). Conversely, doubling the amount of FAP-2286 from 25 μg to 50 μg (i.e., from 17 nmol to 34 nmol) did not significantly enhance gallium incorporation (RCP = 98.60 ± 0.12% vs. 98.12 ± 0.72%, *p* = 0.32) (Figure 6). Consequently, the 25 μg (17 nmol) amount of FAP-2286 was retained, allowing both a high RCP and acceptable specific activity of 7 MBq/nmol.

**FIGURE 7:**
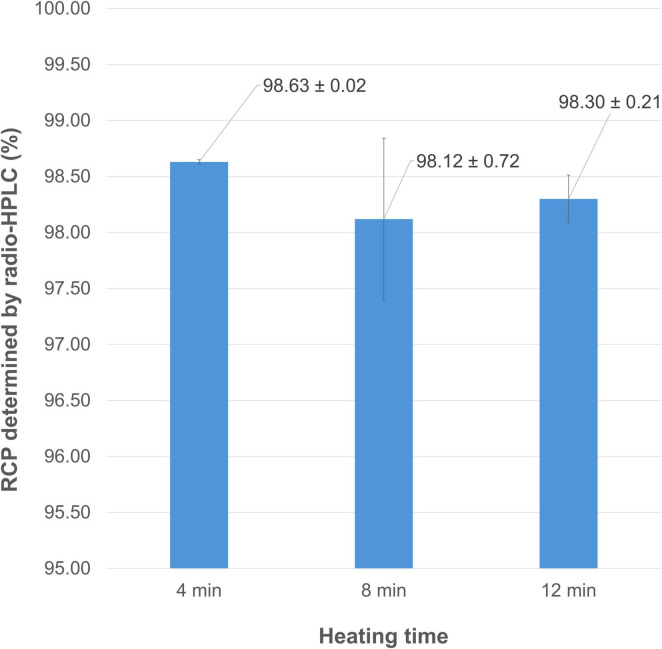
Mean RCP values (determined by radio-HPLC) for [^68^Ga]Ga-FAP-2286 depending on the heating time of the reaction.

Given the short physical half-life of gallium-68, optimizing the heating time required for radiolabeling is also an important consideration. As observed in the study of vector amounts, changing from an 8 to a 12-min reaction time for [^68^Ga]Ga-FAP-2286 preparation did not significantly improve the purity of the final product (RCP = 98.30 ± 0.21% vs. 98.12 ± 0.72%, *p* = 0.7). However, halving the heating time from 8 to 4 min resulted in comparable RCP values (98.63 ± 0.02% vs. 98.12 ± 0.72%, *p* = 0.289) (Figure 7). Consequently, a reduced reaction time of 4 min was adopted to shorten the preparation process.

**FIGURE 8:**
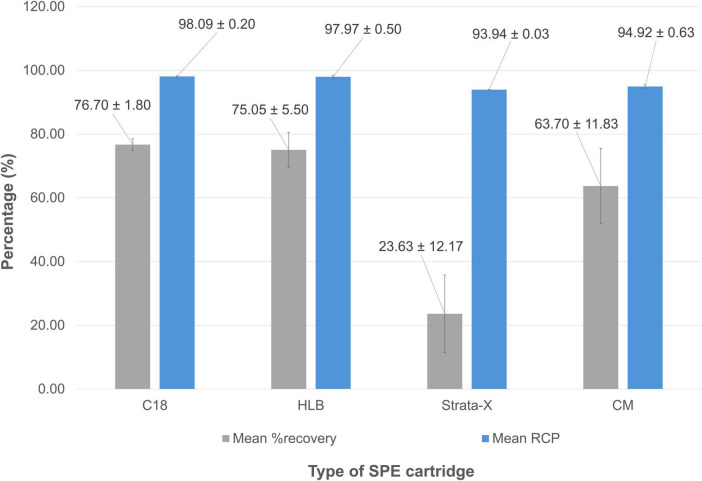
Mean RCP values (determined by radio-HPLC) and mean recovery (%) for [^68^Ga]Ga-FAP-2286 depending on the cartridge used for post-synthesis purification.

Finally, four SPE cartridges were tested for potential implementation as a terminal purification step in the automated [^68^Ga]Ga-FAP-2286 synthesis sequence. Most rely on a “bind and elute” approach, where the crude reaction mixture is loaded onto the stationary phase, small polar impurities are removed by rinsing with WFI, and the product of interest is eluted with an ethanol solution. The CM cartridge is an exception, as its weak cation-exchange properties are designed to retain unreacted ^68^Ga^3+^ while allowing the radiolabeled product to pass through, eliminating the need for an elution step. As shown in Figure 8, all tested purification methods yielded products with good to excellent RCP, ranging from approximately 93 to 98%. However, the Strata-X cartridge (bearing N-vinylpyrrolidone moieties) showed low and variable recovery of the loaded activity, averaging 23.63 ± 12.17%. Similarly, the CM cartridge achieved an average recovery of only 63.7 ± 11.8%. In contrast, apolar-phase cartridges commonly used for ^68^Ga-radiopharmaceutical purification, such as C_18_ and hydrophilic-lipophilic balanced (HLB) cartridges (bearing divinylbenzene-co-N-vinylpyrrolidone moieties), yielded recoveries of approximately 75%. No statistically significant differences were observed between the RCP and recovery values obtained with C_18_ and OASIS HLB cartridges (*p* = 0.716 and *p* = 0.646, respectively), suggesting that either modality could be considered for the terminal purification of [^68^Ga]Ga-FAP-2286. However, it is important to note that this purification step results in the loss of approximately one-quarter of the activity at EoS.

Overall, the optimal radiolabeling conditions included 0.1 M sodium acetate buffer, a high concentration of methionine as an antioxidant agent, 25 μg of FAP-2286, a heating time of 4 min, and terminal purification using a C_18_ or HLB cartridge.

### 3.2 Automated [^68^Ga]Ga-FAP-2286 preparation protocol on GAIA^®^ module

The fully automated synthesis of [^68^Ga]Ga-FAP-2286 using the GAIA^®^ module was completed in around 24 min, from initiation to transfer of the radiolabeled compound into the final product vial. The initial process relied on two sequential “bind and elute” steps (one for concentrating the ^68^Ga-eluate and another for purifying the radiolabeled product) with a 4-min heating phase in between to allow radiolabeling. The activity of the eluate at the time of elution was around 950 MBq. The average molar activity achieved under these conditions was approximately 14.5 MBq/nmol. Notably, incorporating an SCX cartridge minimizes the impact of eluate volume and generator model, potentially enabling the use of eluates from multiple ^68^Ge/^68^Ga generators within a single synthesis (56, 59–61). However, this additional enrichment step of the ^68^Ga eluate required a methodical readjustment of the quantities of buffer solution used in the reaction, in order to correctly control the pH (the volume of saturated sodium chloride pH 1 used to elute the SCX cartridge was maintained at 0.4 mL in all tests). Successive tests have shown that a reaction pH of 3.8 can be achieved using 170 μL of 0.8 M sodium acetate supplemented with 1.03 mL methionine 10 mg/mL. Nevertheless, a significant part of the activity involved in the reaction was found either retained on the SPE cartridge (whether C_18_ or HLB) or in the waste vial. To address this issue and ensure complete complexation of the entire ^68^Ga amount in radiolabeling, the pseudopeptide concentration was doubled, using 50 μg of FAP-2286 in the automated reaction compared to 25 μg in manual assays. Additionally, the heating time was extended to 9 min to optimize the reaction. To ensure complete elution of the SPE cartridge, the volume of ethanol 60% used during terminal purification was increased by 50%, from 1.5 to 2.3 mL. Similarly, the volume of methionine 1 mg/mL in saline was increased proportionally, from 8.6 to 12.8 mL, resulting in a final volume of 15.1 mL and ensuring an ethanol concentration below 9.2% in the terminal formulation.

**FIGURE 9:**
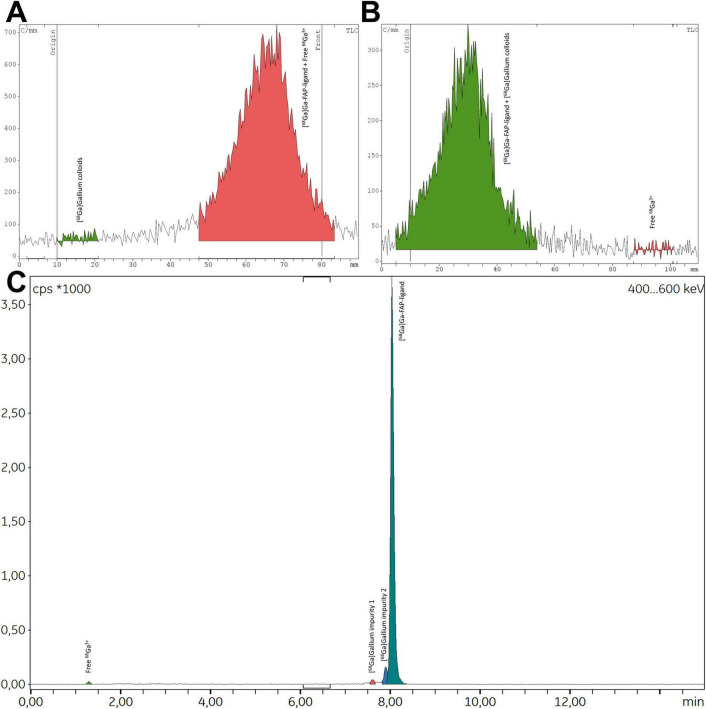
Representative radio-TLC [**(A)** aqueous ammonium acetate 1 M in methanol (1:1); **(B)** aqueous sodium citrate 0.1 M pH 5] and radio-HPLC **(C)** spectra for [^68^Ga]Ga-FAP-2286 produced via automated method.

After the identification of this reliable, secondarily optimized automated protocol, it was implemented for the production of three test batches of [^68^Ga]Ga-FAP-2286. The radiopharmaceutical was obtained with an average RCY of 59.85 ± 3.73% and good purity (RCP > 95%, both in radio-TLC and radio-HPLC, Figure 9). Nevertheless, two radioimpurity peaks were systematically found just before the peak of interest in radio-HPLC, with a significant impact on RCP. As expected, each test synthesis resulted in a clear, colorless final product, with the ^68^Ga radioelement identified by the energy of its gamma photons (peaks at 0.511 and 1.077 MeV) and its half-life (ranging from 61 to 75 min). As anticipated with the use of a pharmaceutical-grade ^68^Ge/^68^Ga generator (62), the radionuclidic purity of the three test batches of [^68^Ga]Ga-FAP-2286 exceeded 99.999%, further enhanced by the final solid-phase purification step. Mean activity at EoS for the three test batches was 517.3 ± 17.8 MBq using a 3-month-old generator. Since the automated synthesis process and the reagents used (buffer and antioxidant) are GMP-compliant, using a pharmaceutical-grade vector would allow the resulting [^68^Ga]Ga-FAP-2286 to be used in a clinical setting.

### 3.3 Transposition of the automated radiolabeling protocol to 3BP-3940

The automated preparation method described above was applied to the synthesis of [^68^Ga]Ga-3BP-3940, another DOTA-pseudopeptide targeting fibroblast activating protein. This transposition was motivated by the high structural homology between FAP-2286 and 3BP-3940, with the latter differing only by the presence of a urea motif replacing an amide function. Three test preparations of ^68^Ga-labeled 3BP-3940 were completed, with extensive quality control providing excellent results, surpassing those obtained for [^68^Ga]Ga-FAP-2286. Specifically, mean RCP was 97.74 ± 1.48% and 97.59 ± 0.93% in radio-TLC and radio-HPLC, respectively. The final formulations displayed a mean activity at EoS of 606.7 ± 44.7 MBq and remained stable over 4 h (RCP > 95.95% in radio-HPLC over this period). The full results of the quality controls performed on the two sets of test batch triplicates are presented in Table 2.

**TABLE 2:** Summary of products specifications for the test batches of [^68^Ga]Ga-FAP-2286 et [^68^Ga]Ga-3BP-3940.

Test	[^68^Ga]Ga-FAP-2286 (*n* = 3)	[^68^Ga]Ga-3BP-3940 (*n* = 3)
Appearance	Clear, colorless solution	Clear, colorless solution
**Identification**		
Energy of gamma photons (MeV)	0.511 and 1.077	0.511 and 1.077
Half-life (min)	68.16 ± 2.71	68.89 ± 1.87
pH	6	6
**Radionuclidic purity**		
(^68^Ga) Gallium (%)	99.99998332 ± 5.46 × 10^–6^	99.99995589 ± 3.02 × 10^–5^
(^68^Ge) Germanium and other γ-emitting impurities (%)	1.67 × 10^–5^ ± 5.46 × 10^–6^	4.41 × 10^–5^ ± 3.02 × 10^–5^
**Radiochemical purity at EoS**		
[^68^Ga]Ga-FAP inhibitor (HPLC)	95.21 ± 0.22	97.59 ± 0.93
[^68^Ga]gallium impurities (HPLC)	4.79 ± 0.22	2.41 ± 0.94
[^68^Ga]Ga-FAP inhibitor (TLC)	96.88 ± 0.71	97.74 ± 1.48
[^68^Ga]gallium impurities (TLC)	3.12 ± 0.71	2.26 ± 1.48
Filter integrity test (mbar)	> 3,500	>3,500
Volume activity at EoS (MBq/mL)*	25.6 ± 0.88	30.0 ± 2.21
Specific activity at EoS (MBq/μg)	9.85 ± 0.35	11.85 ± 0.98
Molar activity at EoS (GBq/μmol)	14.49 ± 0.52	17.44 ± 1.44
Radiochemical yield (based on RCP determined by HPLC)	59.85 ± 3.73	75.62 ± 11.76
Stability over 4 h (HPLC)	≥ 94.98%	≥95.95%

*Calculated with total theoretical volume of 15.2 mL.

## 4 Discussion

FAPI pseudopeptides, such as FAP-2286 and 3BP-3940, are emerging as leading theranostic agents for targeting the tumor microenvironment. Their cyclic pseudopeptide structure provides excellent plasma stability, while their affinity for human FAP reaches nanomolar levels (K_*D*_ = 1.1 nM for FAP-2286) (14). Preliminary evaluations suggest that these compounds offer several advantages over quinoline derivatives such as FAPI-04 and FAPI-46, particularly for targeted radionuclide therapy. However, regarding their diagnostic applications, there are few reports detailing the preparation conditions for [^68^Ga]Ga-FAP-2286 and [^68^Ga]Ga-3BP-3940, whereas the literature on [^68^Ga]Ga-FAPI-46 production is more extensive (52, 59–61, 63–69).

An early report on the preparation of [^68^Ga]Ga-FAP-2286 used a mixture of 1.0 M ammonium acetate and 0.125 M ascorbic acid (4:1) at pH 4.0, already suggesting the interest of an acetate buffer with added antioxidant compound on the overall fate of the reaction (14). Additionally, radiocomplex purification was performed using an OASIS HLB cartridge; however, the preparation process was carried out manually. For the first clinical use of FAP-2286, reported by Baum et al. (25), ^68^Ga-radiotracer preparation was carried out on a synthesis module (Modular-Lab PharmTracer, Eckert & Ziegler). Notably, this configuration allowed to involve up to four generator eluates in the preparation process, achieving an overall activity of up to 2.6 GBq using an SCX cartridge. In relation to these high activities, 150 μg of FAP-2286 were involved in the reaction, leading to maximum specific activities of around 17 MBq/μg. Other radiolabeling reagents were 1 M sodium acetate buffer supplemented with 5 mg of L-ascorbic acid and 1.2 mg of L-methionine, for a total reaction volume of approximately 3.1 mL. These first automated conditions tended to confirm the value of acetate buffers, as well as the benefits of L-methionine in preventing the formation of oxidation byproducts during the preparation of [^68^Ga]Ga-FAP-2286. Several other models of synthesizers were used for the ^68^Ga-radiolabeling of FAP-2286, such as iQS^®^ (ITM Pharma Solutions GmbH) (19) or GRP^®^ (Scintomics Molecular, Applied Theranostics Technologies GmBH) (16), the latter employing conditions previously used for the manual preparation of [^68^Ga]Ga-FAP-2286, namely 1 M ammonium acetate and 200 μL of 0.125 M sodium ascorbate. Recently, a detailed report on the automated synthesis of [^68^Ga]Ga-FAP-2286 on a GRP^®^-3V module was proposed by Hörmann et al., describing an efficient method using HEPES 1.5 M as a buffer (70). As the European Pharmacopoeia classifies HEPES in radiopharmaceutical preparations as an impurity, a maximum quantity of 500 μg per injected volume is permitted in the final formulation. To verify compliance with this limit, chromatographic methods such as TLC (71, 72) or, less commonly, HPLC (73, 74) are recommended. However, this additional quality control step extends the time between radiopharmaceutical production and patient administration. Therefore, despite the excellent buffering properties of HEPES for ^68^Ga-radiolabeling, we opted for low-molarity sodium acetate, with its volume finely adjusted to achieve a pH close to 3.8 after adding 0.4 mL of HCl 0.13 M used to elute the SCX cartridge. Notably, precise control of the buffer volume and molarity during radiolabeling eliminates the need for prior pH adjustment with 30% ultrapure HCl, as is the case here.

Transposing radiolabeling conditions optimized for manual reactions to an automated process often requires adjustments (39), as the best approach would be to study automated radiolabeling conditions directly at the synthesizer scale (63, 75). Nevertheless, the screening of a large number of reaction conditions becomes all the more difficult. In our case, slight adjustments were made to the conditions identified during manual synthesis to suit the fluidic process. In particular, 50 μg of vector was used to enhance reaction completion. These quantities align with several literature protocols, notably for the preparation of [^68^Ga]Ga-3BP-3940 (21, 76, 77). For FAP-2286, Baum’s team reports using 150 μg of pseudopeptide per reaction, which should be considered in conjunction with the combination of eluates from up to four ^68^Ge/^68^Ga generators for a single radiolabeling. This protocol involves a Modular-Lab PharmTracer automaton (Eckert and Ziegler). Under these conditions, molar activities ranged from 11.8 to 25.5 MBq/nmol at elution time (25). Other procedures use 40 μg (17) or even 25 μg of FAP-2286, reaching 20.4–40.8 MBq/nmol and 54.4–65.3 MBq/nmol at elution time, respectively (15). However, the corresponding automated sequences do not include pre-purification of the eluate on SCX cartridges, making these methods less complex. Synthesis using 40 μg vector was performed on an iQS ^68^Ga-fluidic labeling module (ITM Pharma Solutions GmbH). It is worth noting that no protocol for the preparation of [^68^Ga]Ga-3BP-3940 involving pre-treatment of the gallium eluate with a SCX cartridge has yet been reported, and only two have been described for [^68^Ga]Ga-FAP-2286 (25, 70). In all cases, precise pH control within the target range is essential for successful radiolabeling. The heating time of 4 min, sufficient in manual assays to achieve very good RCP, was extended to 9 min, which is still slightly shorter than most reaction times reported in the literature. Indeed, the preparation of [^68^Ga]Ga-FAP-2286 usually requires 10 min (15, 17, 19), or even 15 min heating (14, 78). Only Hörmann et al. describe a reaction time of 6 min at 125°C on a Scintomics GRP-3V module (70). However, given the thermosensitivity of 3BP-3940 demonstrated by Greifenstein’s team (21, 76, 77), such a high temperature was not considered. Instead, a longer radiolabeling time of 9 min at 95°C was preferred. Importantly, no significant side product formation was observed under these conditions, either with FAP-2286 or with 3BP-3940. Similarly, increasing the elution volume from the terminal SPE cartridge aligns with the literature, where reported protocols (when specified) typically indicate a final volume of 15 mL (14, 15, 78) to 17 mL (70) for [^68^Ga]Ga-FAP-2286 preparations. Interestingly, [^68^Ga]Ga-3BP-3940 appears to be more easily eluted from an HLB cartridge using just 0.5 mL of 100% ethanol, enabling a final preparation volume of 10.5 mL (21). Nevertheless, to maintain a single radiolabeling protocol compatible with both pseudopeptides, the use of 2.3 mL of 60% ethanol and 12.8 mL of saline with methionine 1 mg/mL was deemed preferable. This final SPE purification step results in some activity loss on the cartridge and extends the preparation process. However, it ensures the highest purity of the radiolabeled product while also allowing control over the final formulation, especially through the removal of the reaction buffer.

Overall, the method presented here, developed through a thorough study of radiolabeling conditions in manual tests, provides a single turnkey solution for preparing [^68^Ga]Ga-FAP-2286 and [^68^Ga]Ga-3BP-3940 on a GAIA^®^ module. Although this step complicates the process and is likely to have an impact on RCY, the use of a SCX cartridge ensures compatibility with various ^68^Ge/^68^Ga generator models and allows for the potential integration of multiple generators in a single synthesis. This approach enables higher terminal activity, facilitating the management of a larger number of patients. Given the theranostic potential of emerging new-generation FAPI radiopharmaceuticals (79) and the growing innovation in the field of anti-FAP pseudopeptides (80, 81), the demand for PET imaging of the tumor microenvironment is expected to increase significantly.

## Data Availability

The raw data supporting the conclusions of this article will be made available by the author, without undue reservation.
