# Supplementary material for: Specific reaction conditions for efficient automated 68Ga-radiolabeling of the FAP-2286 pseudopeptide on a GAIA® synthesizer
Source: Front Med (Lausanne). 2025 Jul 22;12:1628158. doi: 10.3389/fmed.2025.1628158 (PMC12321554; doi:10.3389/fmed.2025.1628158)
Supplement: Supplementary file 1 [file Data_Sheet_1.docx]

Specific reaction conditions for efficient automated ^68^Ga radiolabeling of the FAP-2286 pseudopeptide on a GAIA^®^ synthesizer

Maissa Ammour^1^, Jade Torchio^1^, Stéphane Renaud^1^, Léa Rubira^1^ and Cyril Fersing^1,2,^*

^1^Nuclear medicine department, Institut régional du Cancer de Montpellier (ICM), Univ. Montpellier, Montpellier, France.

^2^IBMM, Univ Montpellier, CNRS, ENSCM, Montpellier, France.

*** Correspondence:**

Cyril Fersing, PharmD, PhD, Radiopharmacist

Montpellier Cancer Institute, Nuclear Medicine unit,

208 avenue des Apothicaires, 34298 Montpellier Cedex 5 France.

Tel. +33 467612478; Fax. +33 467613059

[cyril.fersing@icm.unicancer.fr](mailto:cyril.fersing@icm.unicancer.fr)

Supplementary Material

**Table of content**

| **1.** | **Detailed automated synthesis sequence for [^68^Ga]Ga-FAP-2286 radiolabeling** | **S2** |
| --- | --- | --- |
| **2.** | **Quality controls for [^68^Ga]Ga-FAP-2286 test batches** | **S4** |
| **3.** | **Quality controls for [^68^Ga]Ga-3BP-3940 test batches** | **S10** |
| **4.** | **Radiochemical stability of the validation batches** | **S16** |

**1. Detailed automated synthesis sequence for [^68^Ga]Ga-FAP-2286 radiolabeling**


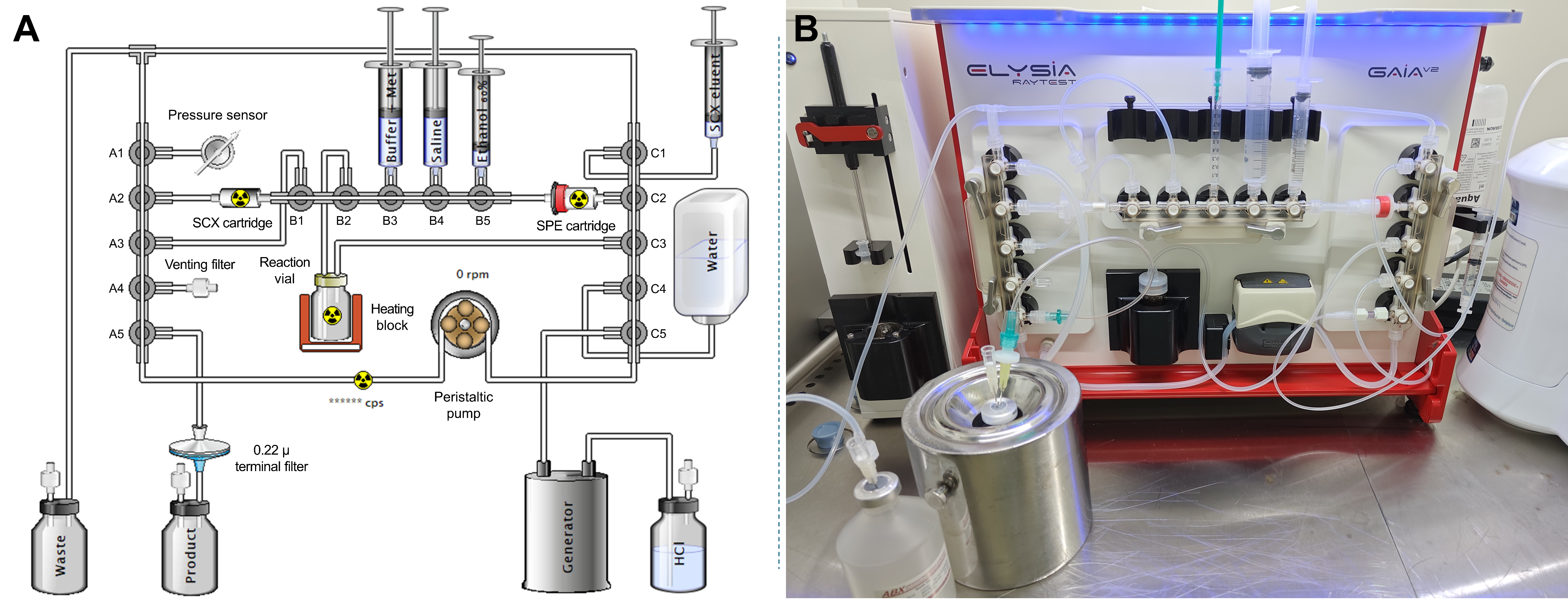


**Fig. S1.** Cassette-based scheme of the GAIA^®^ synthesizer for [^68^Ga]Ga-FAP-2286.


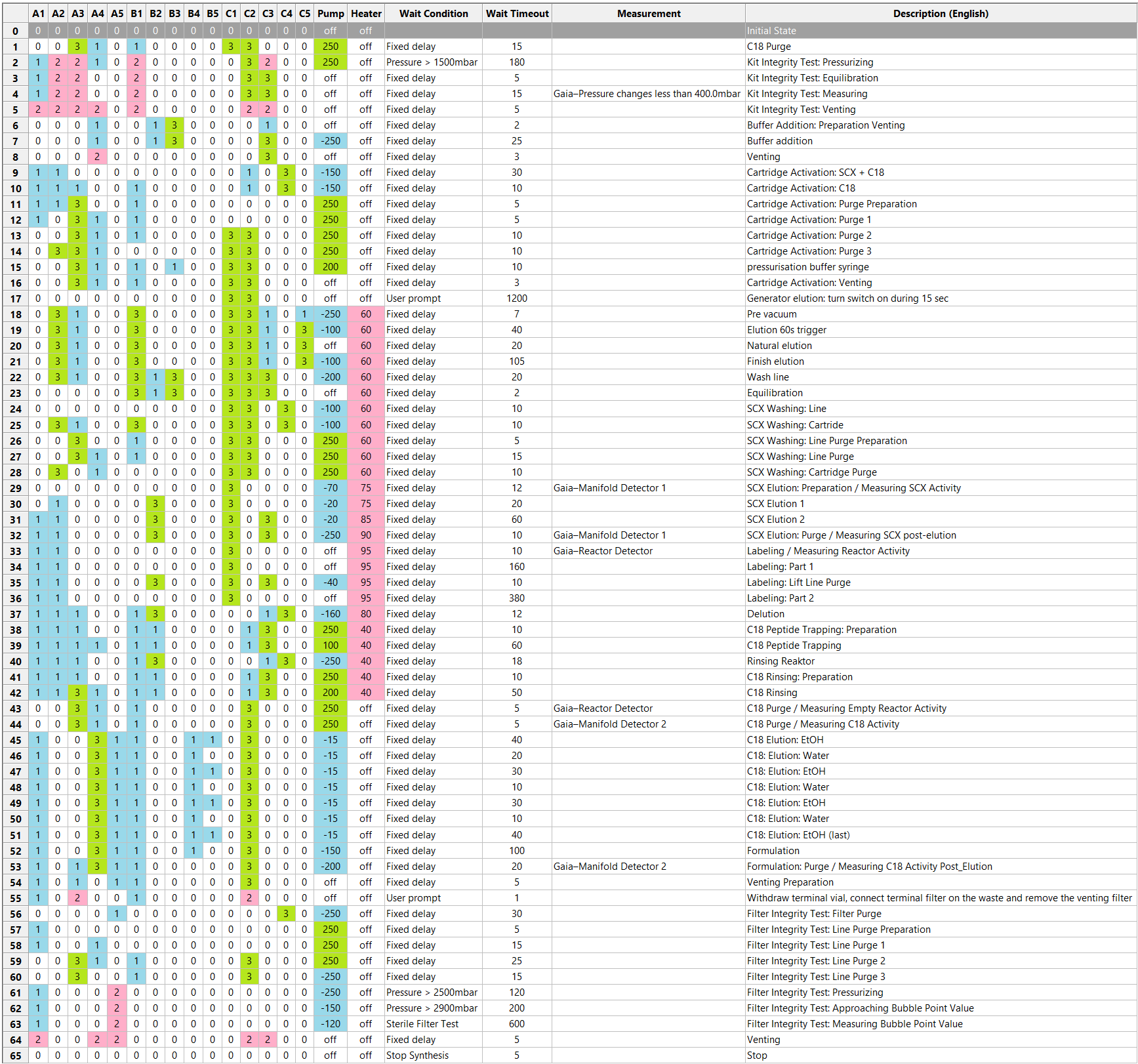


**Fig. S2.** Detailed automated synthesis sequence for [^68^Ga]Ga-FAP-2286 radiolabeling.

**2. Quality controls for [^68^Ga]Ga-FAP-2286 test batches**

**2.1. Radionuclide identity**

**2.1.1. Gamma-spectrometry analyses of the [^68^Ga]Ga-FAP-2286 test batches**

**
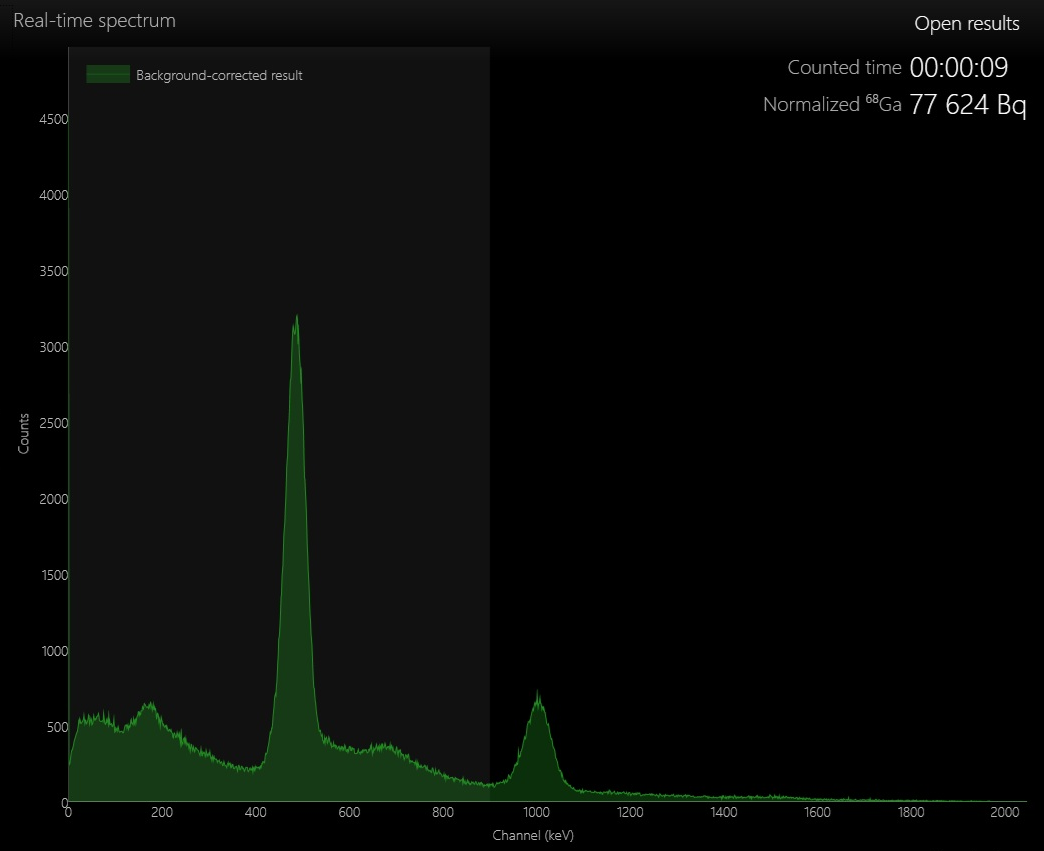
**

**Fig. S3.** Gamma-spectrometry analysis of the [^68^Ga]Ga-FAP-2286 batch 1.


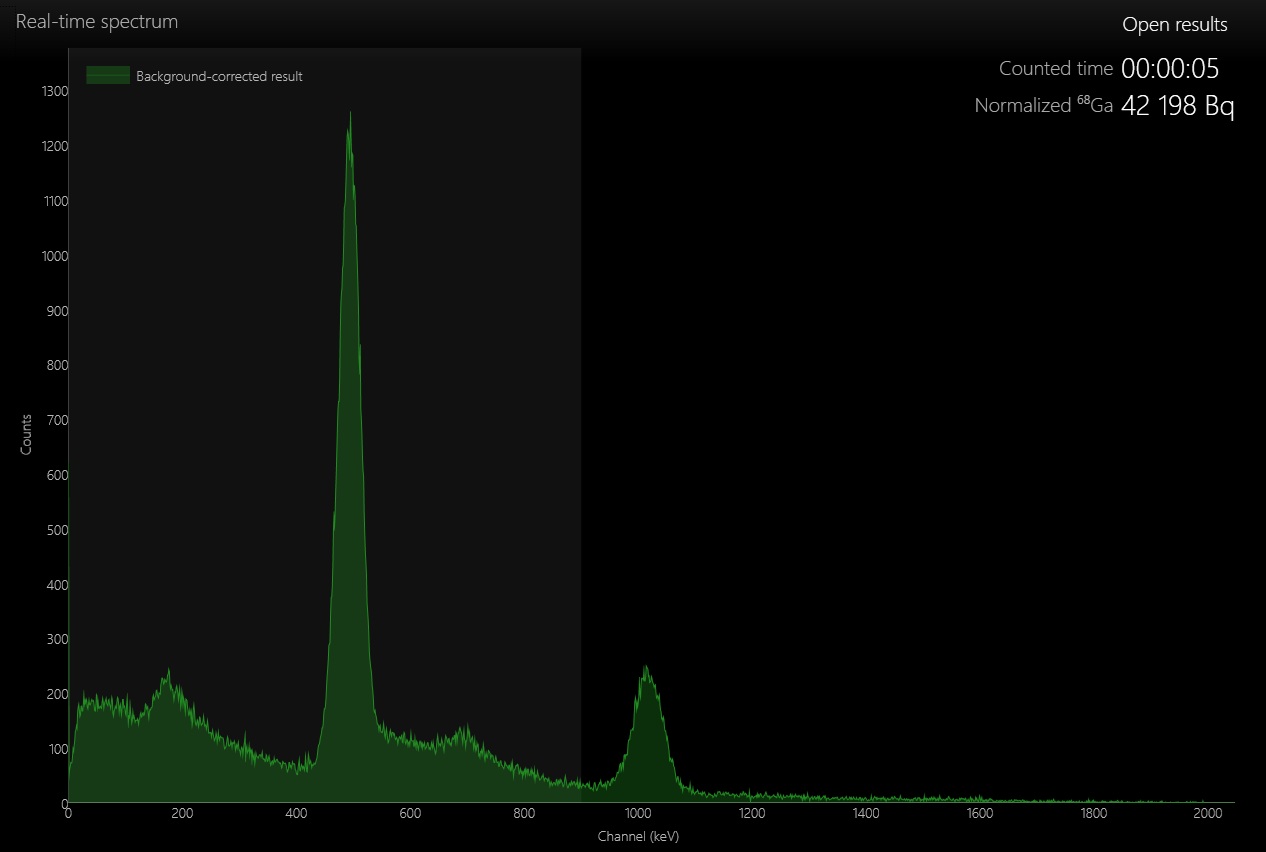


**Fig. S4.** Gamma-spectrometry analysis of the [^68^Ga]Ga-FAP-2286 batch 2.


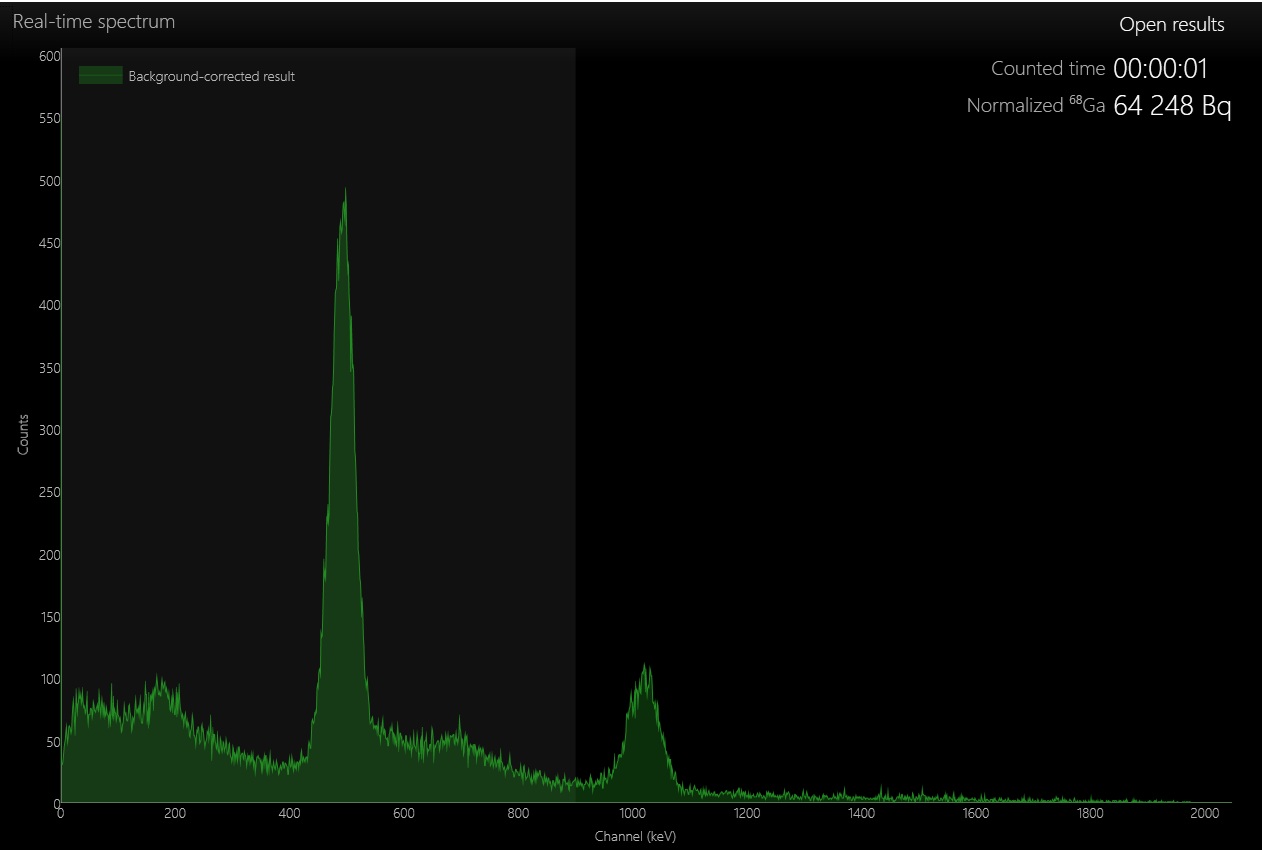


**Fig. S5.** Gamma-spectrometry analysis of the [^68^Ga]Ga-FAP-2286 batch 3.

**2.1.2. Half-life determination for the [^68^Ga]Ga-FAP-2286 test batches**

Batch 1:

| **Time (min)** | **CPM** | **Calculated decay constant (min^-1^)** | **Calculated half-life (min)** |
| --- | --- | --- | --- |
| 0 | 22 515 414 | - | - |
| 4 | 21 512 436 | 0.0113922387 | 60.84380767 |
| 9 | 20 616 063 | 0.0097921791 | 70.78579465 |
| 13 | 19 676 154 | 0.0103686689 | 66.85016058 |
| 18 | 18 872 982 | 0.0098038203 | 70.70174256 |
| 23 | 17 916 476 | 0.0099338872 | 69.77602712 |
| 27 | 17 096 699 | 0.0101968421 | 67.97665121 |
| 32 | 16 456 202 | 0.0097968036 | 70.75238126 |
| 36 | 15 719 496 | 0.0099805116 | 69.45006545 |
| 41 | 15 021 343 | 0.0098714167 | 70.21759909 |
| 45 | 14 370 837 | 0.0099777599 | 69.46921812 |
|  |  |  | **Mean: 68.68**  **± 3.03 min** |

**Fig. S6.** Decay curve for half-life determination of [^68^Ga]Ga-FAP-2286 batch 1.

Batch 2:

| **Time (min)** | **CPM** | **Calculated decay constant (min-1)** | **Calculated half-life (min)** |
| --- | --- | --- | --- |
| 0 | 21 236 525 | - | - |
| 4 | 20 324 531 | 0.0109734991 | 63.16555672 |
| 9 | 19 441 288 | 0.0098137250 | 70.63038588 |
| 13 | 18 404 020 | 0.0110118044 | 62.945831 |
| 18 | 17 710 474 | 0.0100870200 | 68.71674471 |
| 23 | 16 861 370 | 0.0100303204 | 69.10518815 |
| 27 | 16 202 389 | 0.0100208843 | 69.17026081 |
| 32 | 15 621 588 | 0.0095958992 | 72.23368751 |
| 36 | 14 870 788 | 0.0098978840 | 70.02983454 |
| 41 | 14 200 329 | 0.0098160352 | 70.61376272 |
| 45 | 13 531 985 | 0.0100148096 | 69.2122172 |
|  |  |  | **Mean: 68.58**  **± 3.09 min** |

**Fig. S7.** Decay curve for half-life determination of [^68^Ga]Ga-FAP-2286 batch 2.

Batch 3:

| **Time (min)** | **CPM** | **Calculated decay constant (min^-1^)** | **Calculated half-life (min)** |
| --- | --- | --- | --- |
| 0 | 25 398 207 | - | - |
| 5 | 24 192 530 | 0.0097269346 | 71.26059821 |
| 9 | 23 189 956 | 0.0101065919 | 68.58367168 |
| 13 | 22 069 559 | 0.0108061033 | 64.14404544 |
| 18 | 21 039 071 | 0.0104609638 | 66.26035564 |
| 23 | 20 000 130 | 0.0103886873 | 66.72134427 |
| 27 | 19 158 020 | 0.0104428576 | 66.37524037 |
| 32 | 18 331 534 | 0.0101892448 | 68.02733587 |
| 36 | 17 444 229 | 0.0104352695 | 66.42350521 |
| 41 | 16 623 707 | 0.0103377749 | 67.04993924 |
| 45 | 15 984 205 | 0.0102906119 | 67.35723685 |
|  |  |  | **Mean: 67.22 ± 1.85 min** |

**Fig. S8.** Decay curve for half-life determination of [^68^Ga]Ga-FAP-2286 batch 3.

Overall mean of half-life calculated on the 3 batches: **68.16 ± 2.71 min**

**2.2. Radionuclide purity of the [^68^Ga]Ga-FAP-2286 test batches**

|  |  | **Sample activity at t_0_ (MBq)** | **Decay time (min)** | **Gamma counter measurement after decay (CPM)** | **Conversion to activity (Bq) at t_0_;  1 CPM = 0.1377 Bq** | **Calculated radionuclide purity (%)** |
| --- | --- | --- | --- | --- | --- | --- |
| **[^68^Ga]Ga-FAP-2286** | **Batch 1** | 38 | 3436.08 | 29 | 3.9933 | 99.99998943 |
|  | **Batch 2** | 12.8 | 3172.08 | 17 | 2.3409 | 99.99998161 |
|  | **Batch 3** | 21 | 2962.25 | 32 | 4.4064 | 99.99997891 |

**Table S1.** Radionuclide purity of the validation batches.

**2.3. Radiochemical purity of the [^68^Ga]Ga-FAP-2286 test batches**

**
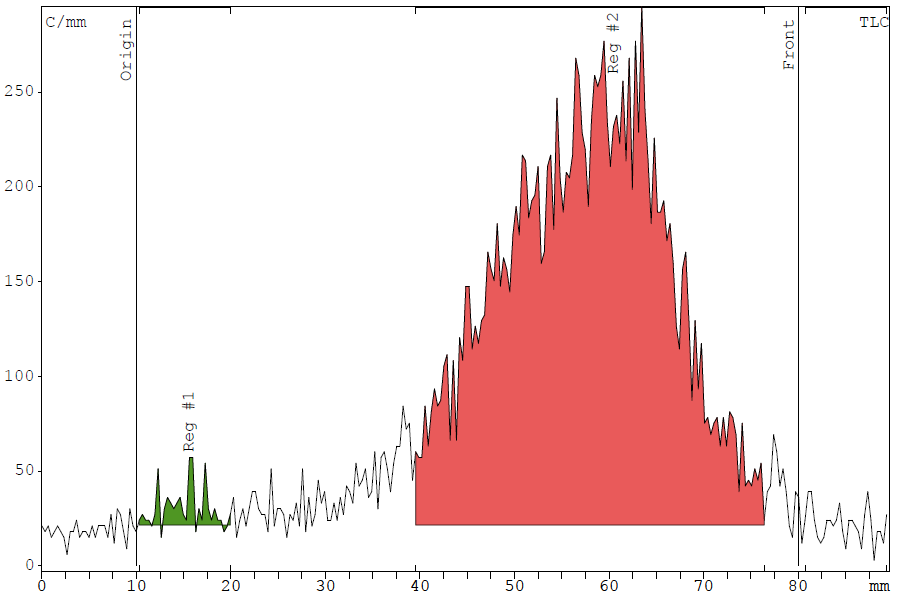

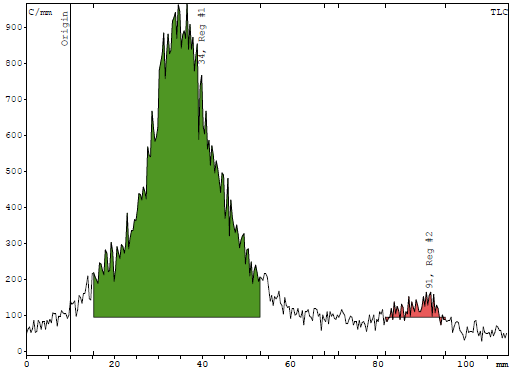
**

**Fig. S9.** Radio-TLC spectra of the [^68^Ga]Ga-FAP-2286 batch 1 at EoS, obtained with aqueous ammonium acetate 1 M in methanol [1:1] (left) and aqueous sodium citrate 0.1 M pH 5 (right). Overall RCP = 96.44%.

**
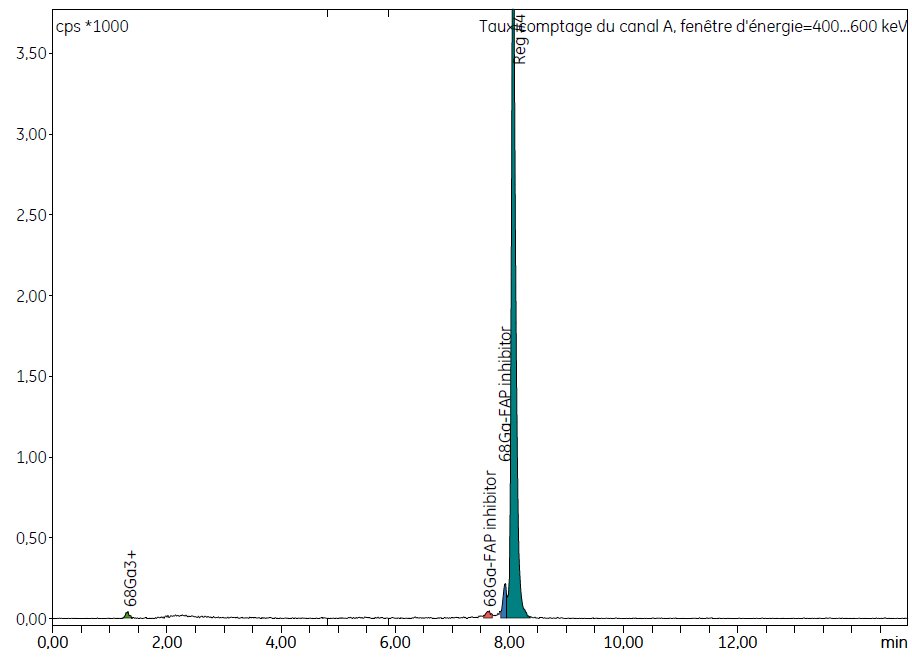
**

68Ga-impurities

**Fig. S10.** Radio-HPLC spectrum of the [^68^Ga]Ga-FAP-2286 batch 1 at EoS. RCP = 95.16%.

**
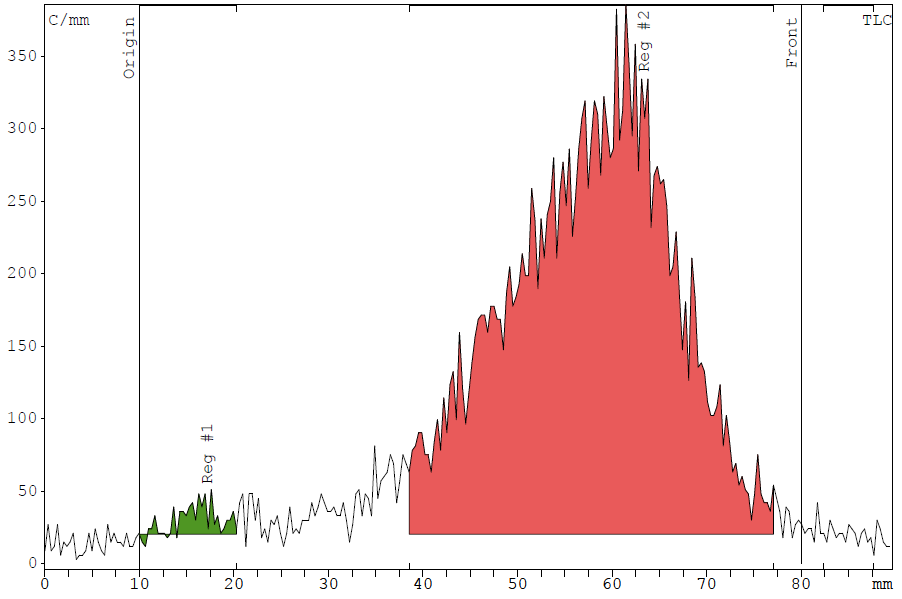
**
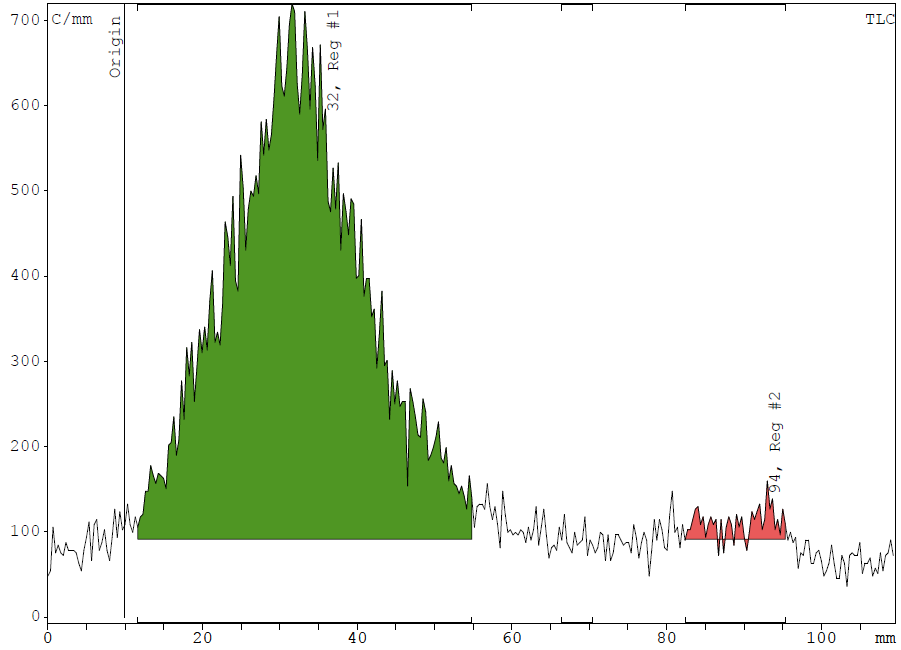


**Fig. S11.** Radio-TLC spectra of the [^68^Ga]Ga-FAP-2286 batch 2 at EoS, obtained with aqueous ammonium acetate 1 M in methanol [1:1] (left) and aqueous sodium citrate 0.1 M pH 5 (right). Overall RCP = 96.5%.

**
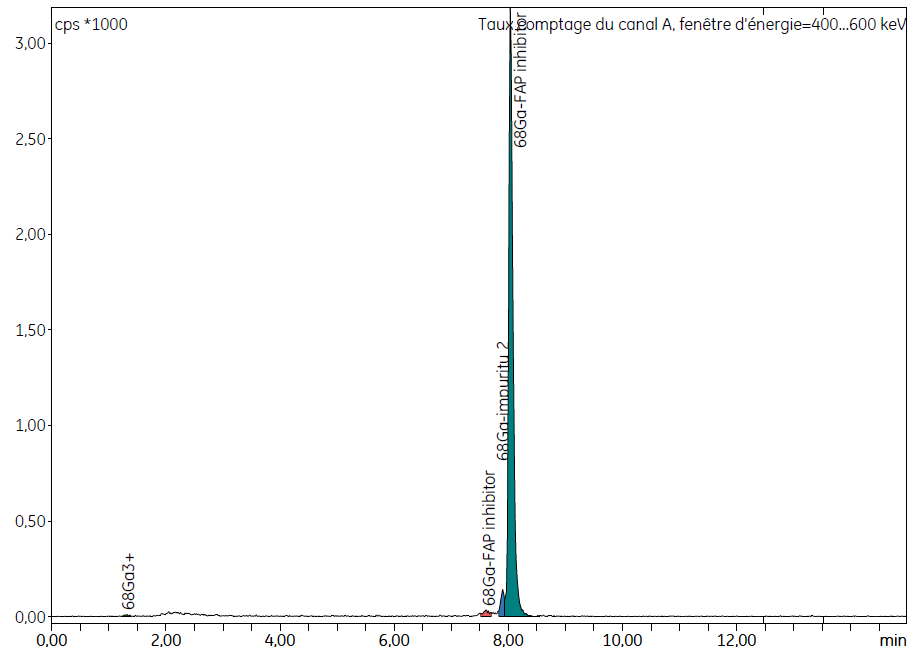
**

68Ga-impurity 1

**Fig. S12.** Radio-HPLC spectrum of the [^68^Ga]Ga-FAP-2286 batch 2 at EoS. RCP = 95.45%.

**
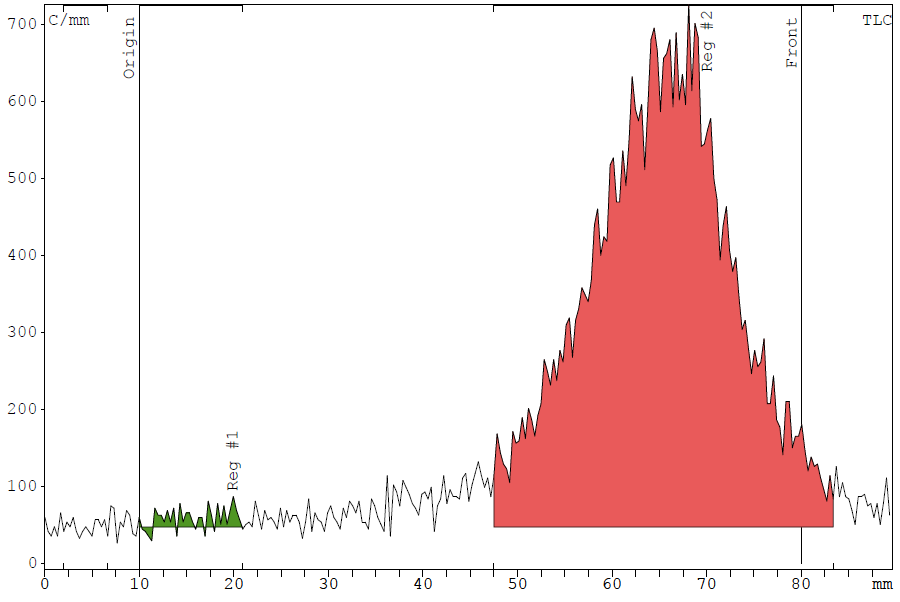
**
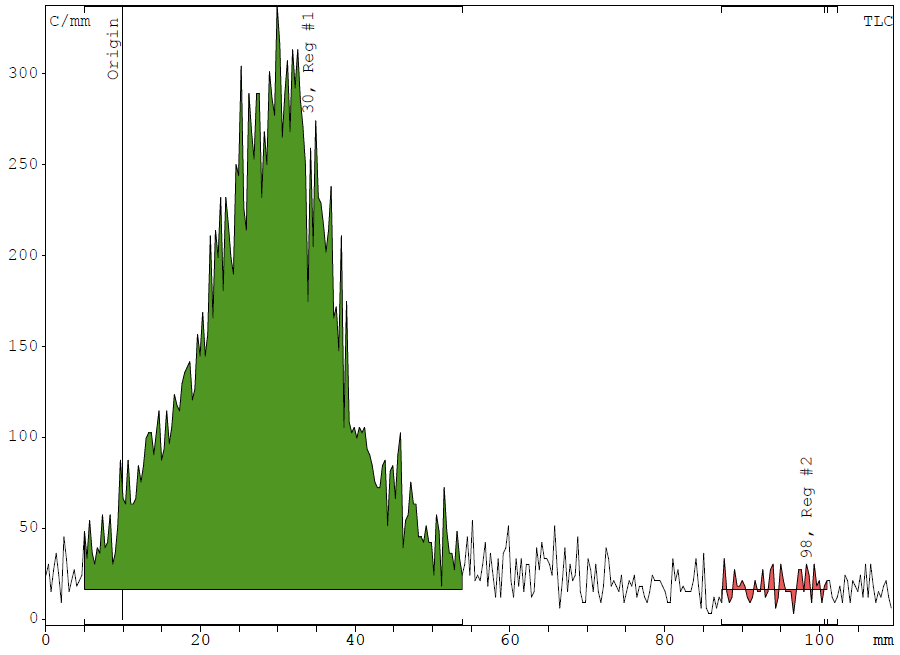


**Fig. S13.** Radio-TLC spectra of the [^68^Ga]Ga-FAP-2286 batch 3 at EoS, obtained with aqueous ammonium acetate 1 M in methanol [1:1] (left) and aqueous sodium citrate 0.1 M pH 5 (right). Overall RCP = 97.7%.

**
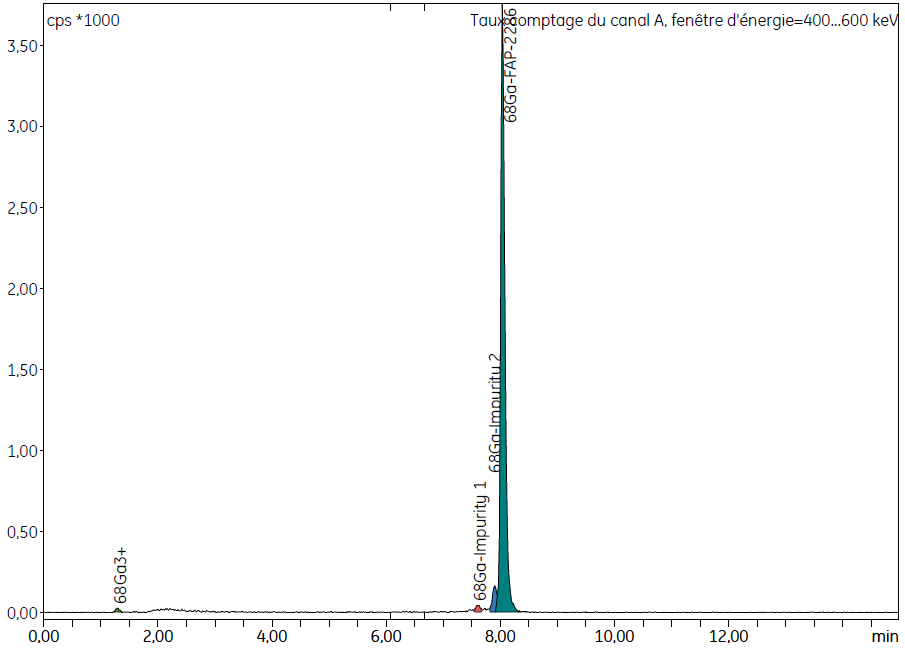
**

**Fig. S14.** Radio-HPLC spectrum of the [^68^Ga]Ga-FAP-2286 batch 3 at EoS. RCP = 95.02%.

**3. Quality controls for [^68^Ga]Ga-3BP-3940**

**3.1. Radionuclide identity**

**3.1.1. Gamma-spectrometry analyses of the [^68^Ga]Ga-3BP-3940 test batches**

**
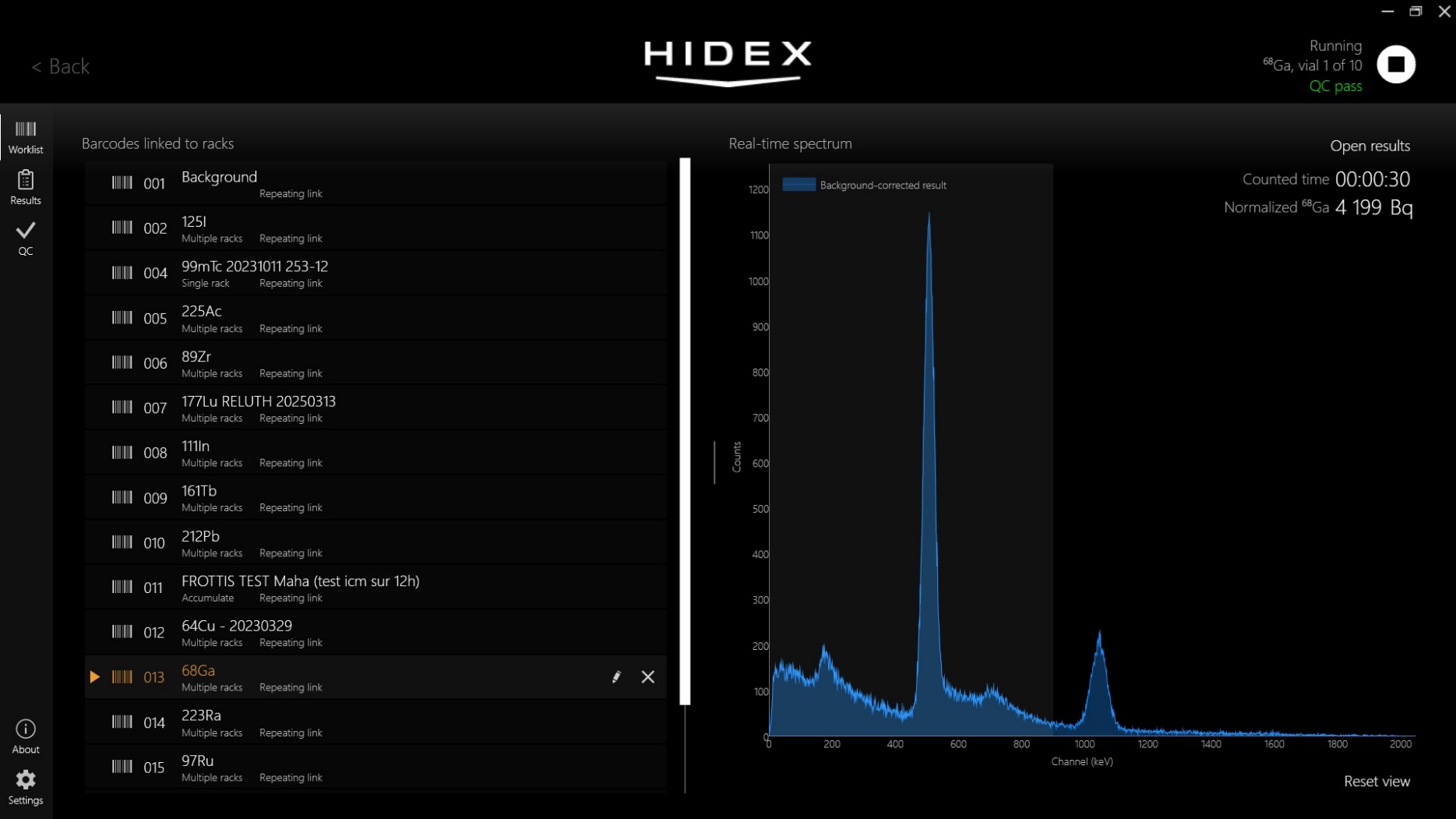
**

**Fig. S15.** Gamma-spectrometry analysis of the [^68^Ga]Ga-3BP-3940 batch 1.


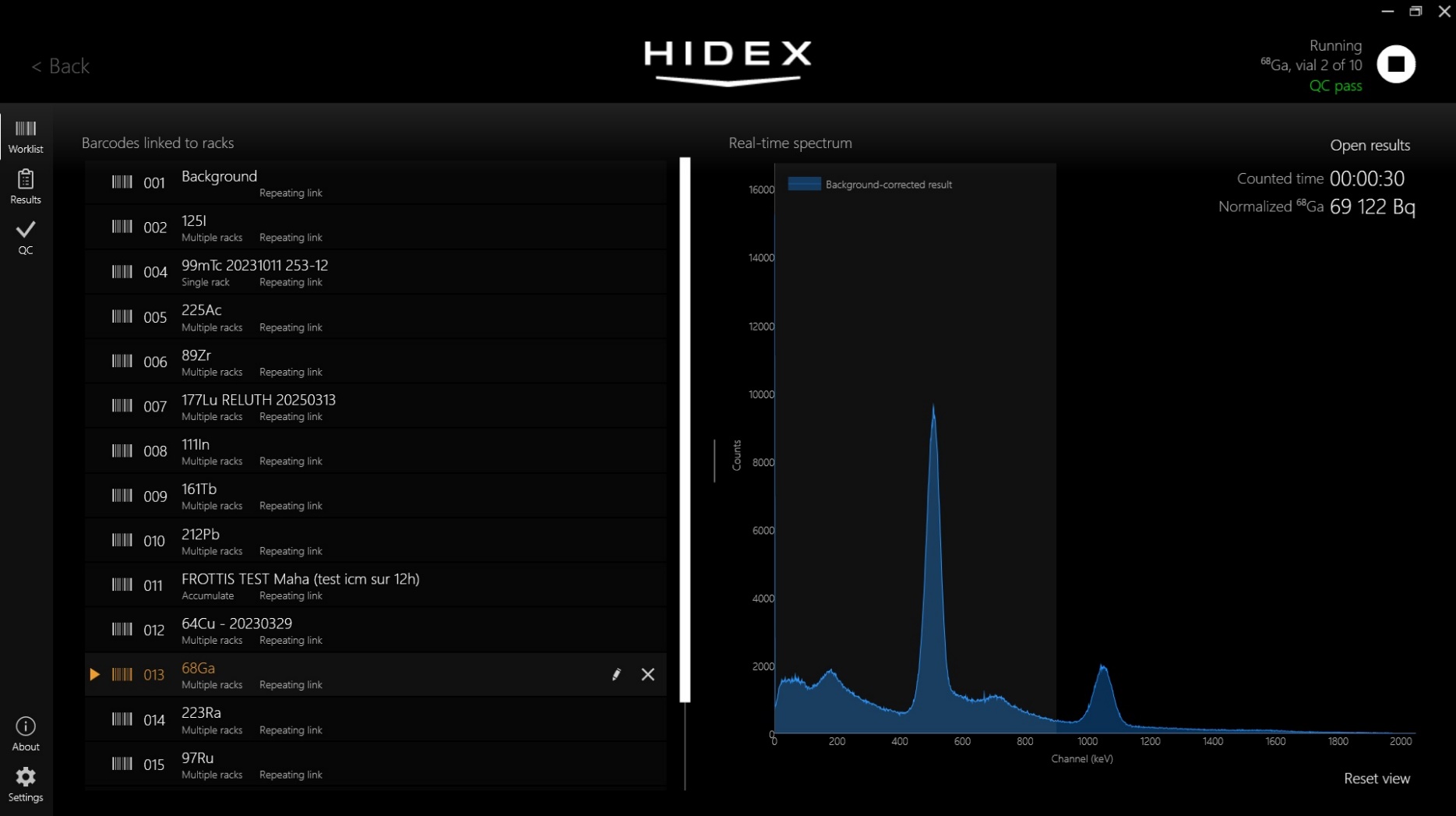


**Fig. S16.** Gamma-spectrometry analysis of the [^68^Ga]Ga-3BP-3940 batch 2.


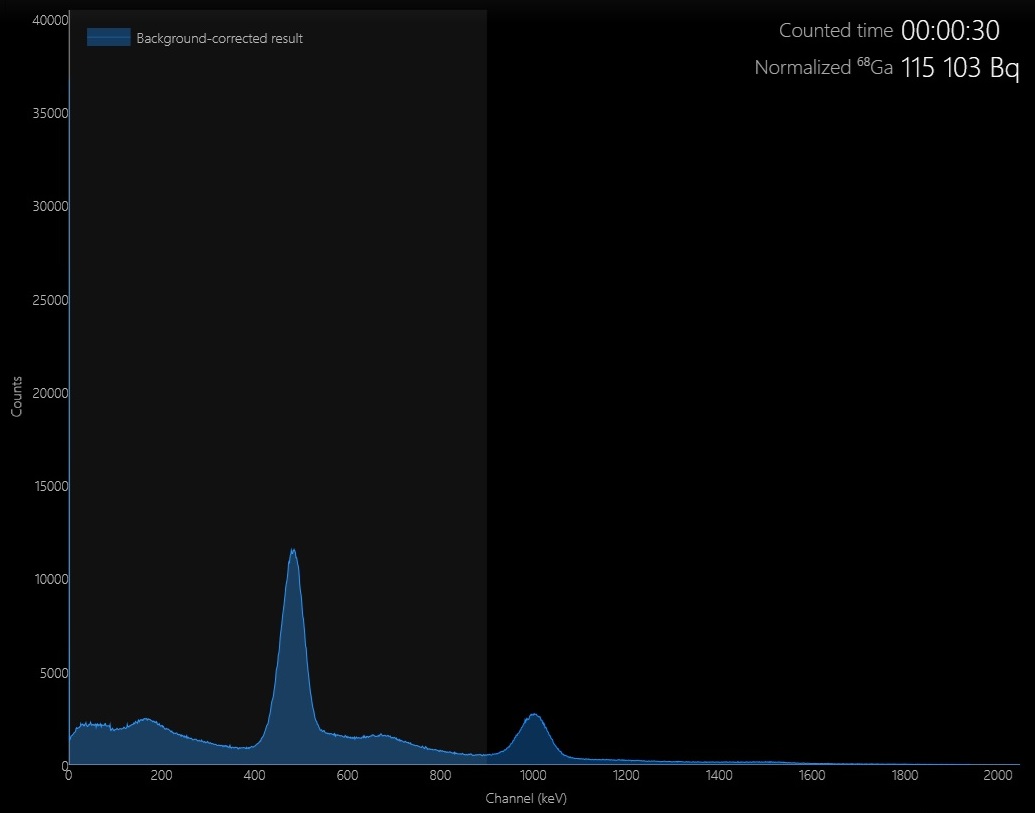


**Fig. S17.** Gamma-spectrometry analysis of the [^68^Ga]Ga-3BP-3940 batch 3.

**3.1.2. Half-life determination for the [^68^Ga]Ga-3BP-3940 test batches**

Batch 1:

| **Time (min)** | **CPM** | **Calculated decay constant (min-1)** | **Calculated half-life (min)** |
| --- | --- | --- | --- |
| 0 | 251 923 | - | - |
| 11 | 227 837 | 0.0095499212 | 72.58145581 |
| 21 | 204 306 | 0.0100165959 | 69.19987483 |
| 31 | 185 473 | 0.0097794977 | 70.87758488 |
| 42 | 165 576 | 0.0100606642 | 68.8967615 |
| 52 | 148 945 | 0.0100902870 | 68.69449612 |
| 62 | 133 802 | 0.0101280226 | 68.4385501 |
| 73 | 119 794 | 0.0101996627 | 67.95785346 |
| 83 | 108 850 | 0.0100782800 | 68.77633696 |
| 94 | 97 919 | 0.0100867642 | 68.7184873 |
| 104 | 87 502 | 0.0101578063 | 68.23788119 |
|  |  |  | **Mean: 69.24**  **± 1.42 min** |

**Fig. S18.** Decay curve for half-life determination of [^68^Ga]Ga-3BP-3940 batch 1.

Batch 2:

| **Time (min)** | **CPM** | **Calculated decay constant (min-1)** | **Calculated half-life (min)** |
| --- | --- | --- | --- |
| 0 | 4 147 313 | - | - |
| 11 | 3 715 200 | 0.0104560500 | 66.29149468 |
| 21 | 3 355 801 | 0.0101249137 | 68.45956408 |
| 31 | 3 024 667 | 0.0100811562 | 68.75671452 |
| 42 | 2 713 332 | 0.0101706950 | 68.15140758 |
| 52 | 2 450 491 | 0.0101023034 | 68.61278595 |
| 62 | 2 205 259 | 0.0101096685 | 68.56280022 |
| 73 | 1 987 460 | 0.0100933689 | 68.67352073 |
| 83 | 1 790 875 | 0.0100855296 | 68.72689947 |
| 94 | 1 609 884 | 0.0101008075 | 68.62294725 |
| 104 | 1 449 827 | 0.0100958927 | 68.65635397 |
|  |  |  | **Mean: 68.35**  **± 0.74 min** |

**Fig. S19.** Decay curve for half-life determination of [^68^Ga]Ga-3BP-3940 batch 2.

Batch 3:

| **Time (min)** | **CPM** | **Calculated decay constant (min^-1^)** | **Calculated half-life (min)** |
| --- | --- | --- | --- |
| 0 | 19 913 924 | - | - |
| 4 | 19 035 922 | 0.0112728399 | 61.48824819 |
| 9 | 18 258 907 | 0.0096406855 | 71.89812159 |
| 13 | 17 430 970 | 0.0102438982 | 67.66439573 |
| 18 | 16 679 458 | 0.0098467380 | 70.39358454 |
| 22 | 15 993 673 | 0.0099648172 | 69.55944747 |
| 26 | 15 402 845 | 0.0098794982 | 70.16016069 |
| 31 | 14 654 256 | 0.0098931736 | 70.06317805 |
| 35 | 14 020 762 | 0.0100251416 | 69.1408871 |
| 40 | 13 424 054 | 0.0098592753 | 70.30406976 |
| 44 | 12 893 472 | 0.0098795011 | 70.16013996 |
|  |  |  | **Mean: 69.08 ± 2.87 min** |

**Fig. S20.** Decay curve for half-life determination of [^68^Ga]Ga-3BP-3940 batch 3.

Overall mean of half-lives calculated for the 3 batches: **68.89 ± 1.87 min**

**3.2. Radionuclide purity of the [^68^Ga]Ga-3BP-3940 test batches**

|  |  | **Sample activity at t_0_ (MBq)** | **Decay time (min)** | **Gamma counter measurement after decay (CPM)** | **Conversion to activity (Bq) at t_0_;  1 CPM = 0.1377 Bq** | **Calculated radionuclide purity (%)** |
| --- | --- | --- | --- | --- | --- | --- |
| **[^68^Ga]Ga-3BP-3940** | **Batch 1** | 1.8 | 6853.25 | 13 | 1.8 | 99.9998993 |
|  | **Batch 2** | 30.7 | 6774.25 | 47 | 6.5 | 99.9999787 |
|  | **Batch 3** | 19 | 3854.06 | 13 | 10.1 | 99.9999468 |

**Table S2.** Radionuclide purity of the validation batches.

**3.3. Radiochemical purity of the [^68^Ga]Ga-3BP-3940 test batches**

**
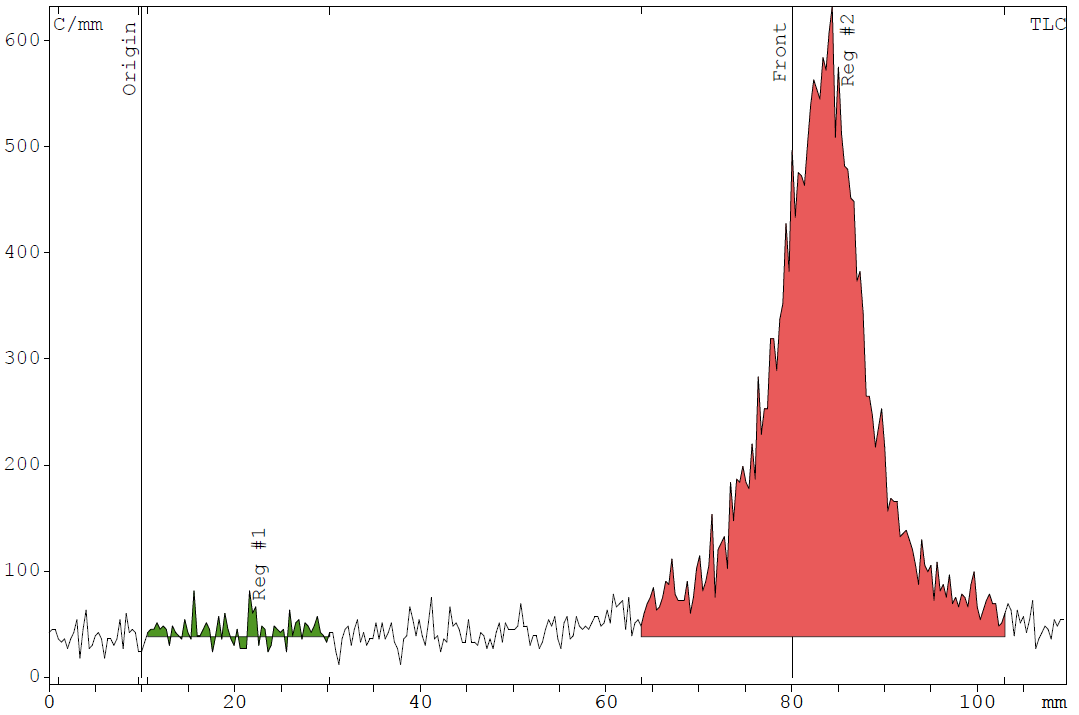

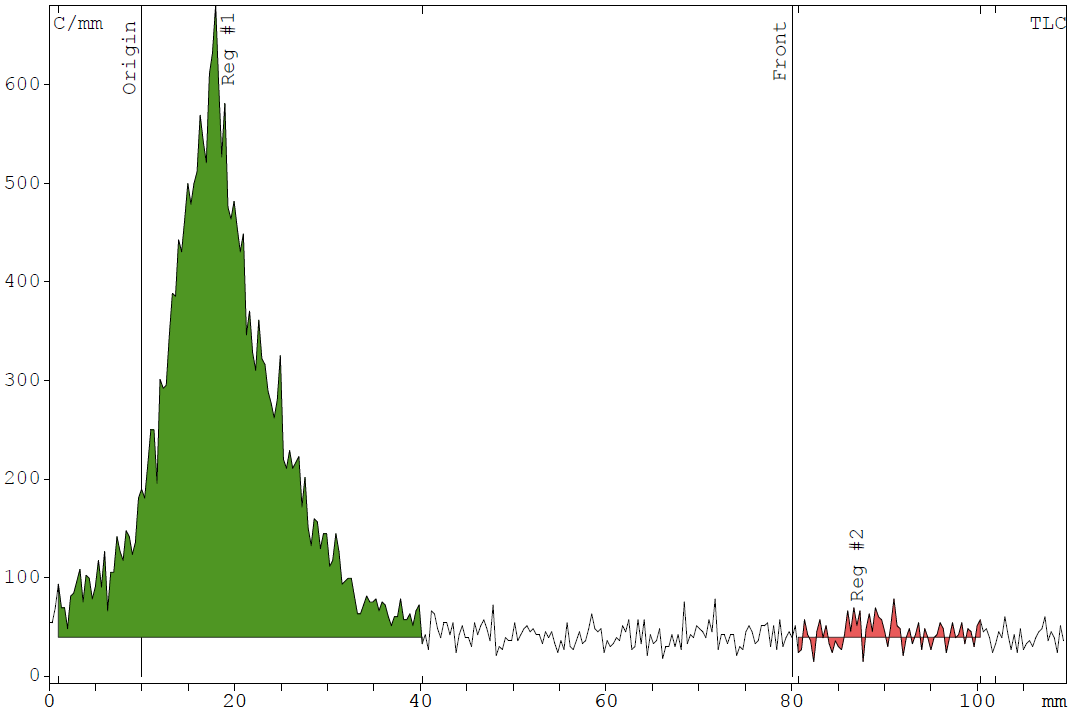
**

**Fig. S21.** Radio-TLC spectra of the [^68^Ga]Ga-3BP-3940 batch 1 at EoS, obtained with aqueous ammonium acetate 1 M in methanol [1:1] (left) and aqueous sodium citrate 0.1 M pH 5 (right). Overall RCP = 97.24%.

**
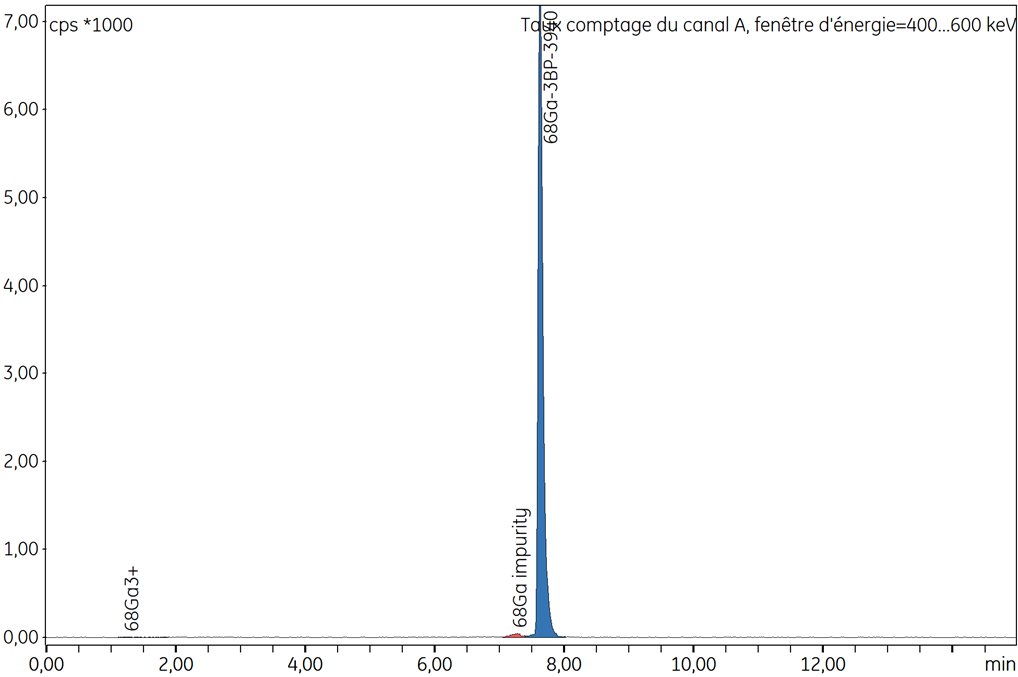
**

**Fig. S22.** Radio-HPLC spectrum of the [^68^Ga]Ga-3BP-3940 batch 1 at EoS. RCP = 98.23%.

**
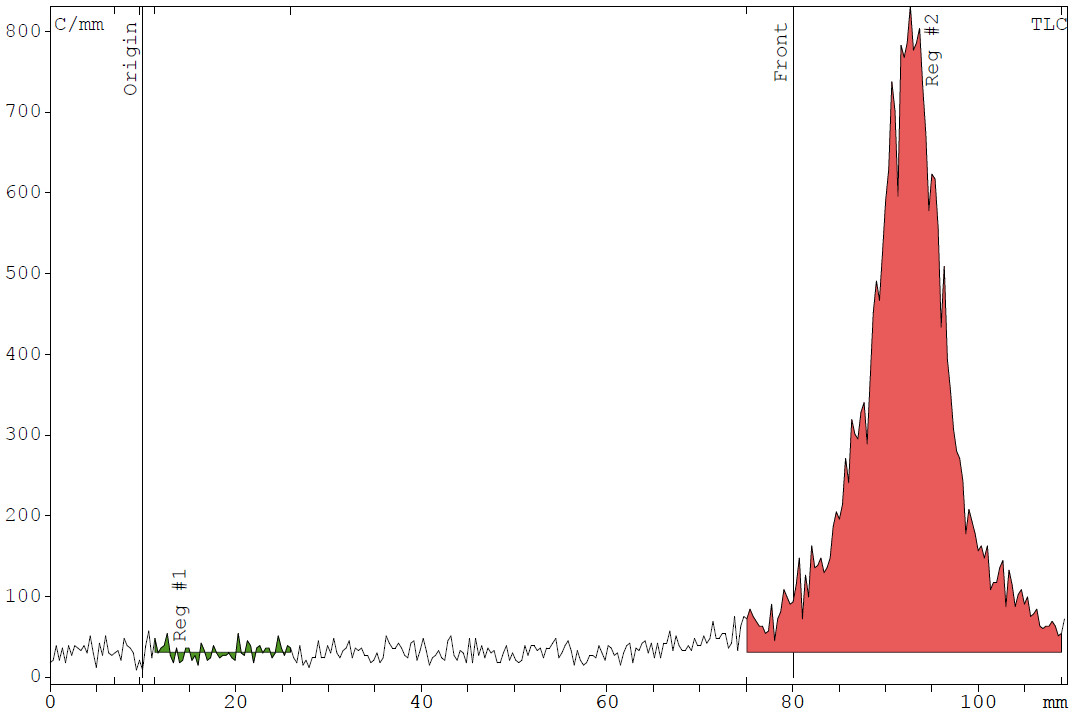
**
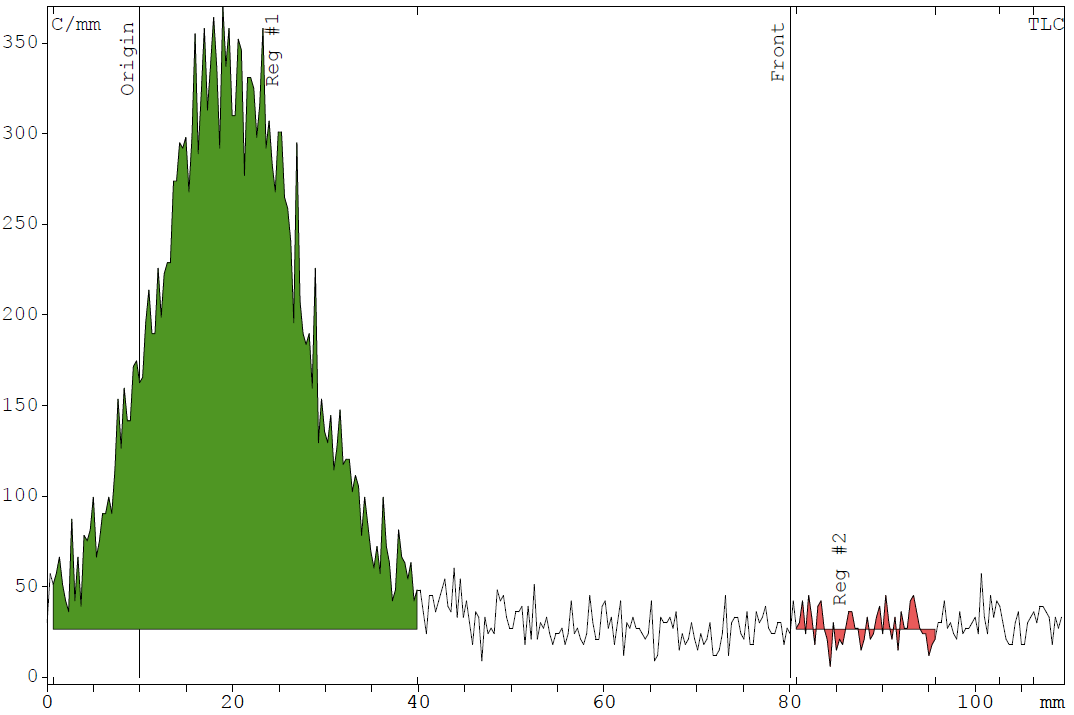


**Fig. S23.** Radio-TLC spectra of the [^68^Ga]Ga-3BP-3940 batch 2 at EoS, obtained with aqueous ammonium acetate 1 M in methanol [1:1] (left) and aqueous sodium citrate 0.1 M pH 5 (right). Overall RCP = 99.41%.


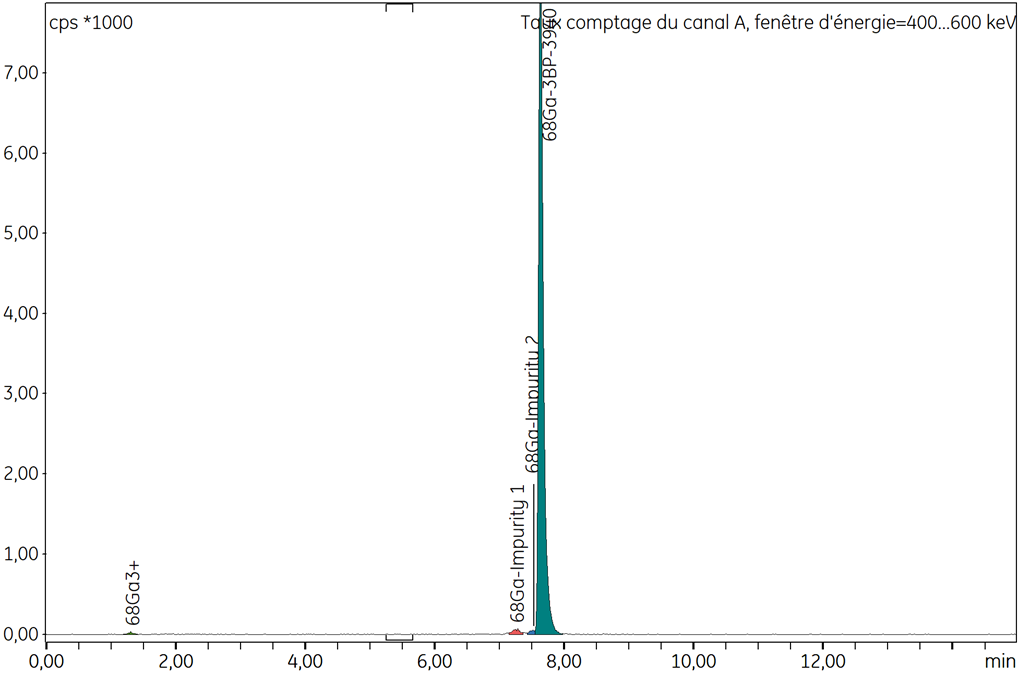


**Fig. S24.** Radio-HPLC spectrum of the [^68^Ga]Ga-3BP-3940 batch 2 at EoS. RCP = 98.03%.

**
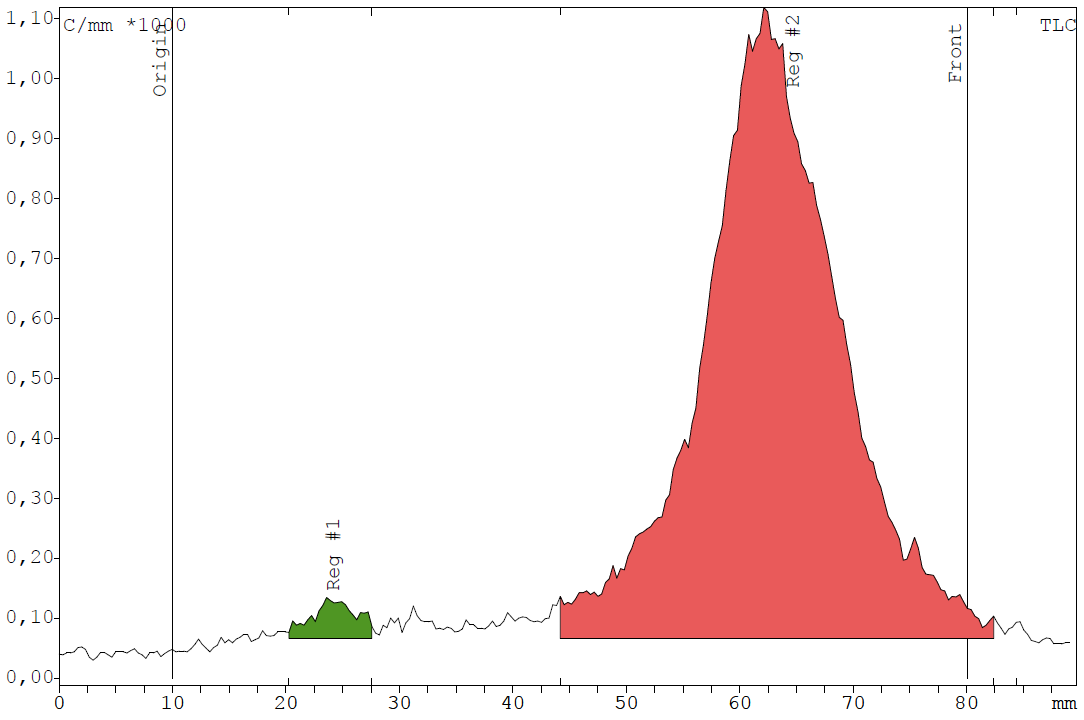
**
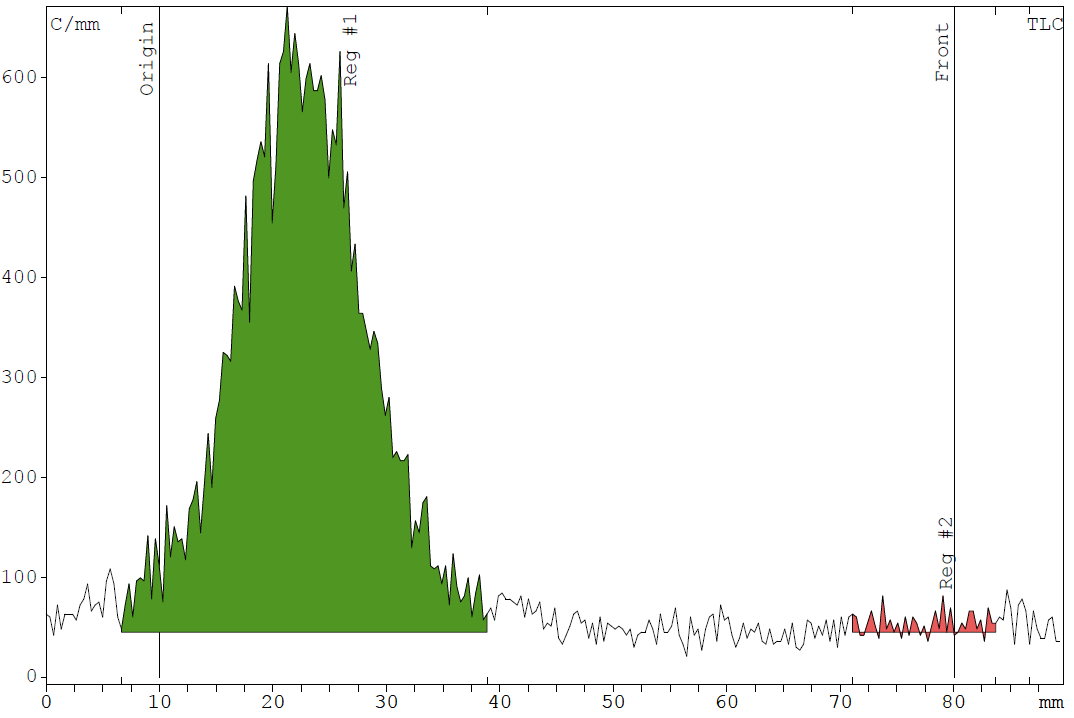


**Fig. S25.** Radio-TLC spectra of the [^68^Ga]Ga-3BP-3940 batch 3 at EoS, obtained with aqueous ammonium acetate 1 M in methanol [1:1] (left) and aqueous sodium citrate 0.1 M pH 5 (right). Overall RCP = 96.58%.


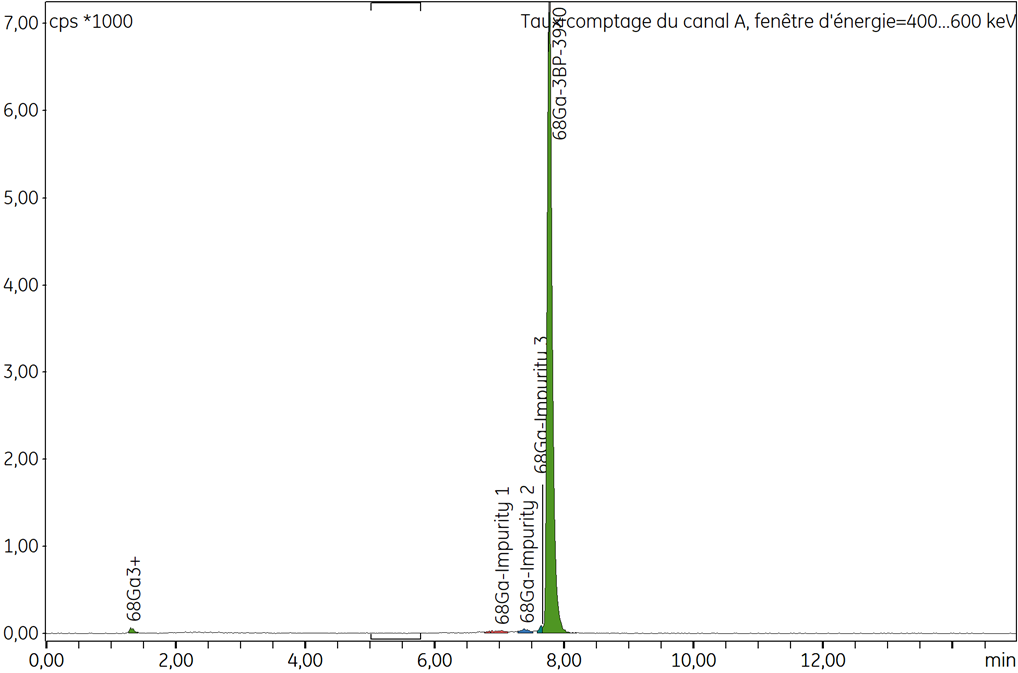


**Fig. S26.** Radio-HPLC spectrum of the [^68^Ga]Ga-3BP-3940 batch 3 at EoS. RCP = 96.52%.

**4. Radiochemical stability of the validation batches determined on radio-HPLC**

| **[^68^Ga]Ga-FAP-2286** | **Time of measurement after radiolabeling (h)** | | | | |
| --- | --- | --- | --- | --- | --- |
|  | **0.50** | **1.00** | **2.00** | **3.00** | **4.00** |
| Batch 1 | 95.16 | 95.18 | 95.06 | 95.1 | 95.03 |
| Batch 2 | 95.45 | 95.12 | 95.39 | 95.32 | 95.58 |
| Batch 3 | 95.02 | 94.98 | 95.05 | 95.08 | 95.12 |
| %SD | 95.21 | 95.09 | 95.17 | 95.17 | 95.24 |
| Mean | 0.22 | 0.10 | 0.19 | 0.13 | 0.30 |
| **[^68^Ga]Ga-3BP-3940** |  |  |  |  |  |
| Batch 1 | 98.23 | 98.06 | 98.05 | 97.85 | 98.09 |
| Batch 2 | 98.03 | 98.11 | 97.62 | 97.95 | 97.99 |
| Batch 3 | 96.52 | 95.95 | 96.14 | 96.80 | 96.45 |
| %SD | 97.59 | 97.37 | 97.27 | 97.53 | 97.51 |
| Mean | 0.93 | 1.23 | 1.00 | 0.64 | 0.92 |

**Table S3.** Radiochemical stability of the test batches determined by radio- HPLC.

**Fig. S27.** Mean radiochemical stability over time of [^68^Ga]Ga-FAP-2286 (green) and [^68^Ga]Ga-3BP-3940 (blue) test batches determined by radio-HPLC.
